# Supplementary material for: Vitamin D deficiency, impaired lung function and total and respiratory mortality in a cohort of older men: cross-sectional and prospective findings from The British Regional Heart Study
Source: BMJ Open. 2021 Dec 21;11(12):e051560. doi: 10.1136/bmjopen-2021-051560 (PMC8693094; doi:10.1136/bmjopen-2021-051560)
Supplement: Supplementary data [file bmjopen-2021-051560supp001.pdf]

Supplementary Table S1 Lung function and adjusted hazard ratios (95%CI) for total mortality according to 5 groups of 25(OH)D.

			**25(OH)D (ng/ml)**		
	<10 (N=363)<br>(N=363)	10-19 (N=1499)	29-29<br>(N=1207)	30-39<br>(N=403)	≥40<br>(N=103)
**Lung function**					
**Mean (Std)**					
FEV1(L)	2.35 (0.71)	2.58 (0.65)	2.68 (0.65)	2.66 (0.62)	2.64 (0.63)
FVC (L)	3.17 (0.90)	3.36 (0.85)	3.47 (0.83)	3.48 (0.81)	3.43 (0.88)
FEV1/FVC	0.74 (0.13)	0.77 (0.12)	0.77 (0.11)	0.77 (0.12)	0.77 (0.11)
%COPD	32.5	22.0	20.8	20.8	24.3
% Severe COPD	10.7	3.9	3.6	3.2	5.8
% Restrictive	28.7	30.2	26.8	25.3	28.1
**Total mortality**					
Rate/1000 per-yrs (n)	67.9 (287)	46.4 (988)	41.0 (740)	41.4 (251)	38.4 (61)
Hazard ratio (95%CI)					
Age+season adjusted	1.91 (1.44,2.53)	1.00	1.23 (0.95,1.59)	1.04 (0.79,1.36)	1.00 (0.76,1.33)
+Adjusted	1.52 (1.14,2.02)	1.00	1.14 (0.88,1.48)	1.01 (0.78,1.33)	0.97 (0.72,1.26)

+Adjusted for age, season, smoking, physical activity, social class, diabetes, use of antihypertensive treatment, pre-existng CVD and BMI.
