# Supplementary material for: Vitamin D deficiency, impaired lung function and total and respiratory mortality in a cohort of older men: cross-sectional and prospective findings from The British Regional Heart Study
Source: BMJ Open. 2021 Dec 21;11(12):e051560. doi: 10.1136/bmjopen-2021-051560 (PMC8693094; doi:10.1136/bmjopen-2021-051560)
Supplement: Author's manuscript [file bmjopen-2021-051560.draft_revisions.pdf]

BMJ Open

BMJ Open is committed to open peer review. As part of this commitment we make the peer review history of every article we publish publicly available.

When an article is published we post the peer reviewers' comments and the authors' responses online. We also post the versions of the paper that were used during peer review. These are the versions that the peer review comments apply to.

The versions of the paper that follow are the versions that were submitted during the peer review process. They are not the versions of record or the final published versions. They should not be cited or distributed as the published version of this manuscript.

BMJ Open is an open access journal and the full, final, typeset and author-corrected version of record of the manuscript is available on our site with no access controls, subscription charges or pay-per-view fees (http://bmjopen.bmj.com).

If you have any questions on BMJ Open's open peer review process please email info.bmjopen@bmj.com

BMJ Open

Vitamin D deficiency, impaired lung function and total and respiratory mortality in older men: The British Regional Heart Study

Journal:	*BMJ Open*
Manuscript ID	bmjopen-2021-051560
Article Type:	Original research
Date Submitted by the Author:	22-Mar-2021
Complete List of Authors:	Wannamethee, Goya; UCL Medical School, UCL Department of Primary Care & Population Health<br>Welsh, Paul; University of Glasgow, Institute of Cardiovascular and Medical Sciences<br>Papacosta, Olia; UCL Medical School, Primary Care and Population Health<br>Lennon, Lucy; University College London Medical School, Primary Care and Population Health<br>Whincup, Peter; St George's University of London
Keywords:	EPIDEMIOLOGY, RESPIRATORY MEDICINE (see Thoracic Medicine), NUTRITION & DIETETICS

SCHOLARONE™
Manuscripts

I, the Submitting Author has the right to grant and does grant on behalf of all authors of the Work (as defined in the below author licence), an exclusive licence and/or a non-exclusive licence for contributions from authors who are: i) UK Crown employees; ii) where BMJ has agreed a CC-BY licence shall apply, and/or iii) in accordance with the terms applicable for US Federal Government officers or employees acting as part of their official duties; on a worldwide, perpetual, irrevocable, royalty-free basis to BMJ Publishing Group Ltd ("BMJ") its licensees and where the relevant Journal is co-owned by BMJ to the co-owners of the Journal, to publish the Work in this journal and any other BMJ products and to exploit all rights, as set out in our licence.

The Submitting Author accepts and understands that any supply made under these terms is made by BMJ to the Submitting Author unless you are acting as an employee on behalf of your employer or a postgraduate student of an affiliated institution which is paying any applicable article publishing charge ("APC") for Open Access articles. Where the Submitting Author wishes to make the Work available on an Open Access basis (and intends to pay the relevant APC), the terms of reuse of such Open Access shall be governed by a Creative Commons licence – details of these licences and which Creative Commons licence will apply to this Work are set out in our licence referred to above.

Other than as permitted in any relevant BMJ Author's Self Archiving Policies, I confirm this Work has not been accepted for publication elsewhere, is not being considered for publication elsewhere and does not duplicate material already published. I confirm all authors consent to publication of this Work and authorise the granting of this licence.

For peer review only

Vitamin D deficiency, impaired lung function and total and respiratory mortality in older men: The British Regional Heart Study

S Goya Wannamethee[1], Paul Welsh[2], Olia Papacosta[1], Lucy Lennon[1] ,Peter H Whincup[4]

1.UCL Department of Primary Care & Population Health, UCL Medical School, Rowland Hill Street, London. NW3 2PF UK

2. Institute of Cardiovascular & Medical Sciences, BHF Glasgow Cardiovascular Research Centre, University of Glasgow

3. Population Health Research Institute, St George's, University of London, Cranmer Terrace, London. SW17 0RE, UK

Address for Correspondence Prof S Goya Wannamethee, Department Primary Care and Population Health, UCL Medical School, Royal Free Campus, Rowland Hill St, London NW32PF, UK.

e-mail g.wannamethee@ucl.ac.uk

Word count text: 3153

Tables 5

CONFLICT OF INTEREST NONE

For peer review only

ABSTRACT

Objectives Vitamin D deficiency is associated with chronic obstructive pulmonary disease (COPD). We examined the association between circulating 25-hydroxyvitamin D (25OHD) and lung function impairment and assessed whether vitamin D deficiency is related to long term mortality in those with impaired lung function.

Design Prospective study

Setting General practices in the UK.

Participants 3575 men aged 60-79 years with no prevalent HF.

Outcome measures Airway obstruction and mortality. The Global Initiative on Obstructive Lung diseases (GOLD) spirometry criteria was used to define airway obstruction.

Results During the follow-up period of 20 years, there were 2327 deaths (114 COPD deaths). Vitamin D deficiency was defined as serum 25OHD levels <10 ng/ml; insufficiency as 25OHD 10-19 ng/mL; normal as 25OHD >20 ng/mL. Vitamin D deficiency was associated with moderate COPD (FEV/FVC <70% and FEV1 50-80%) and severe COPD (FEV/FVC <70% and FEV1 < 50%) but not with mild COPD (FEV/FVC <70% and FEV1 > 80%) or restrictive lung disease (FEV1/FVC >70% and FVC <80%). However, vitamin D deficiency was associated with increased risk of total and respiratory mortality in both men with COPD and men with restrictive lung disease after adjustment for confounders and inflammation. The adjusted HRs (95% CI) for total mortality comparing levels of 25OHD <10ng/mL to 25OHD >20 ng/ml were 1.39 (1.10,1.75), 1.52 (1.17,1.98), 1.58 (1.17,2.14) and 1.39 (0.83,2.33) for those with no lung impairment, restrictive lung function, mild/moderate COPD and severe COPD respectively.

For peer review only

Conclusion Our findings suggest that vitamin D supplementation trials in older adults with impaired lung function are needed to assess the impact on health outcomes.

Key words: Vitamin D (25OHD); chronic obstructive lung disease; lung function ; mortality

For peer review only

Strengths and limitations of this study

- This study is carried out in a high risk group for vitamin D deficiency and COPD and examined restrictive and obstructive lung disease separately.

- The study population is socially representative of the UK.

- The study took into account a wide range of confounders and inflammatory markers that could explain the associations between vitamin D and lung function and mortality.

-. The study is limited by the use of a single measurement of vitamin D.

- The study is restricted to a predominantly white male population of European extraction,

For peer review only

INTRODUCTION

Chronic obstructive lung disease (COPD) is a common chronic inflammatory disease in the elderly and is a major cause of morbidity and mortality [1]. Much attention has focussed on identifying factors which may influence prognosis and mortality in these patients. Vitamin D deficiency (ascertained from measuring the circulating 25-dehydroxyvitamin D [25OHD] metabolite) is recognised as an important health problem particularly in the elderly [2] and has been associated with various diseases including respiratory infections, autoimmune disease, CVD, diabetes and cancer [3-6]. Although vitamin D deficiency is traditionally known for its role in bone health, there is growing interest in the role of vitamin D in the pathogenesis and severity of COPD [7]. Observational studies have found Vitamin D deficiency to be highly prevalent in patients with COPD [8-12]. While many cross-sectional population studies have associated higher 25OHD with better lung function as measured by the FEV1 [10-16], prospective studies of the association of baseline 25OHD with lung function decline and incident COPD have produced conflicting results [9-11,15,16]. Whether the association between 25OHD and impaired lung function is specific to COPD is also unclear. Vitamin D deficiency has been associated with increased mortality in the general population [17-19] but the associations with pulmonary death and total mortality in COPD patients remains uncertain. Longitudinal studies that have examined the association between vitamin D and mortality in COPD patients have mostly reported no significant association with mortality [20-24]. However, most of these previous studies have targeted moderate and severe COPD patients only. Two recent prospective studies showed that vitamin D deficiency or low vitamin D was associated with all-cause mortality in those with COPD in the general population [25,26] . Few studies have examined the association between circulating vitamin D deficiency and impaired lung function in the general older population specifically and whether vitamin D deficiency is associated with mortality in older adults

For peer review only

with milder degrees of impaired lung function (restrictive or obstructive) has not been well studied. The aim of the present study is to 1) investigate the association between vitamin D deficiency and both restrictive and obstructive lung function impairment and 2) whether vitamin D deficiency is related to long term mortality in those with impaired lung function.

SUBJECTS AND METHODS

The data that support the findings of this study are available from the corresponding author upon reasonable request (contact Lucy Lennon at l.lennon@ucl.ac.uk).

The British Regional Heart Study is a prospective study involving 7735 men aged 40-59 years drawn from one general practice in each of 24 British towns, who were screened between 1978 and 1980 [27]. The population studied was socio-economically representative of British men and comprises predominantly white Europeans (>99%). In 1998-2000, all surviving men, then aged 60-79 years, were invited for a 20th year follow-up examination. Ethical approval was obtained from all relevant local research ethics committees. All men completed a mailed questionnaire providing information on their lifestyle and medical history, had a physical examination and provided a fasting blood sample. The samples were frozen and stored at -20^{0}C on the day of collection and transferred in batches for storage at -70°C until analysis, carried out after no more than one freeze-thaw cycle. The men were asked whether a doctor had ever told them that they had angina or MI, heart failure (HF) or stroke; details of their medications were recorded at the examination including use of bronchodilators (BNF code 3.1). 4252 men (77% of available survivors) attended for examination.

<u>Study subjects</u>

For peer review only

Blood measurements of vitamin D, were available in 3754 men (88%) at the 20 year follow-up examination (1998-2000). Of these men, we excluded 88 men with a history of a diagnosis of HF as these men have exceptional mortality rates and reduced lung function and a further 91 men with no lung function measures leaving 3575 men for analysis.

Cardiovascular risk factor measurements at 1998-2000

Anthropometric measurements including body weight, height and waist circumference (WC) were carried out. Details of measurement and classification methods for smoking status, physical activity, social class, alcohol intake, blood pressure and blood lipids in this cohort have been described [28,29]. Interleukin-6 was assayed using a high-sensitivity ELISA (R & D Systems, Oxford, UK). C-reactive protein (CRP) was assayed by ultra sensitive nephelometry (Dade Behring, Milton Keynes, UK).

Vitamin D

Measurement of 25OHD was performed on EDTA-anticoagulated plasma via a high-throughput method for the measurement of $25OHD_3$ and $-D_2$ using a gold-standard liquid chromatography-tandem mass spectrometry method following an automated solid-phase extraction procedure [30,31]. Our method is calibrated and controlled using reagents from Chromsystems GmbH (Manchester, UK) and is currently in routine clinical use. The lower limit of sensitivity was 4ng/ml for both $25OHD_3$ and total $25OHD_2$ .Results are reported as total 25OHD [$25OHD_2$ + $25OHD_3$); virtually all participants had an undetectable $25OHD_2$, which is commensurate with results observed in routine NHS use. Vitamin D deficiency was defined as 25OHD <10 ng/ml] and vitamin D insufficiency as 25OHD 10-19 ng/ml [2,31,32].

For peer review only

Lung function

Forced expiratory volume in 1 second (FEV1) and forced vital capacity (FVC) were measured using a Vitalograph Compact spirometer with the subject seated [29]. We used predictive equations for FEV1 and FVC that were derived from the general population Health Survey for England for males aged >25 years [33]. The following equations were used:

predicted FEV1=-exp[-9.37674+0.000183*age-0.00011*age^2+log (height)]

predicted FVC= exp[-10.36706+0.00434*age-0.00011*age^2+log(height)]

Predicted FEV1%=raw FEV1/predicted FEV1 and predicted FVC%=raw FVC/predicted FVC. We divided the men into 5 lung pattern groups according to their FEV1/FVC ratio using a modification of the criteria developed by GOLD for defining the severity stages of obstructive airways [34]: 1) severe airflow obstruction (FEV1/FVC <0.70 and FEV1 <50% predicted), moderate airflow obstruction (FEV1/FVC <0.70 and FEV1 ≥ 50 to <80%), mild airflow obstruction (FEV1/FVC <0.70 and FEV1≥ 80%), restricted (FEV1/FVC >0.70 and FVC <80% predicted) and normal (FEV1/FVC >0.70 and FVC >80% and not on bronchodilators).

Follow-up

All men have been followed up from initial examination (1978-1980) for cardiovascular morbidity [27] and follow-up has been achieved for 99% of the cohort. In the present analyses, all-cause mortality is based on follow-up from re-examination in 1998-2000 at mean age 60-79 years to September 2019. Information on death was collected through the established "tagging" procedures provided by the National Health Service registers. COPD

For peer review only

deaths were defined as deaths due to chronic bronchitis (ICD-9 codes 490-491), emphysema (ICD-9 code 492), and chronic airway obstruction (ICD-9 code 496).

Statistical analysis

The men were classified into 3 vitamin D groups: 25OHD <10ng/ml (deficient), 10-19 ng/ml (insufficient) and ≥ 20 ng/ml (normal). Cox's proportional hazards model was used to assess the multivariate-adjusted hazards ratio (relative risk) in a comparison of three vitamin D groups: <10ng/ml, 10-19 ng/ml and ≥20ng/ml and as well as in a 1 unit increase in 25(OH)D. In multivariate analyses, smoking, social class, physical activity, alcohol intake, diabetes, pre-existing CVD were fitted as categorical variables; IL-6 was fitted as fitted as a continuous variable.

Patients and public involvement

Patients and the public were not involved in the conduct, design, analysis and interpretation of this research.

RESULTS

During the mean follow-up period of 20 years there were 2327 deaths from all causes in the 3575 men with no diagnosed HF, including 114 deaths due to COPD.

Baseline characteristics

For peer review only

Vitamin D deficiency was significantly associated with a more adverse health profile including increased smoking, heavy drinking, physical inactivity, obesity, hypertension and inflammation (CRP, IL-6) (Table 1). Lung function (both FEV and FVC) improved with increasing vitamin D levels. Men with vitamin D deficiently had the highest rates of COPD seen for moderate and severe COPD but not mild COPD. There was no association between vitamin D deficiency and prevalence of restrictive lung impairment.

Restrictive and obstructive lung function patterns and vitamin D

Vitamin D was significantly lower in men with moderate or severe airway obstruction but not in those with mild airway obstruction compared to those with normal function (Table 2). Moderate and severe COPD but not restrictive lung function patterns were associated with increased odds of having vitamin D deficiency after adjustment for potential confounders including age, season, smoking, physical activity, social class, BMI, diabetes, renal dysfunction and pre-existing CVD (Table 2).

Vitamin D levels and Total mortality

Vitamin D was significantly and inversely associated with total mortality even after adjustment for potential confounders and inflammation (IL-6) (Table 3). The inverse association persisted upon exclusion of current smokers [adjusted HR=1.41 (1.19,1.68) and 1.11 (1.00,1.24)] for those with vitamin D deficiency and insufficiency respectively (p=0.002 for trend). The inverse association was seen for respiratory and COPD mortality but not CVD mortality after adjustment (Table 3).

For peer review only

When examined by lung function status, vitamin D deficiency was associated with significantly increased mortality in those with normal lung function as well as in those with restrictive lung disorder or mild/moderate COPD even after adjustment for confounders and inflammation (Table 4). In men with severe COPD a non- significant increase in mortality was seen possibly due to small numbers. Vitamin D deficiency was associated with increased risk of respiratory mortality even after adjustment although this was significant only in the restrictive and mild and moderate COPD group possibly because of small numbers (Table 5). The number of COPD deaths was small but vitamin D deficiency was associated with increased risk of COPD mortality in all groups with the exception of those with severe COPD and this was significant in those with mild moderate COPD.

Discussion

Our findings confirm previous observational population studies on the association between low 25OHD and COPD and total mortality [13-19] and extend these findings further. The association was not explained by smoking, physical activity or inflammation (IL-6 or CRP) which are known to relate to both COPD and circulating vitamin D levels [35]. Restrictive lung function, by contrast, was not associated with vitamin D deficiency. We have shown that 25OHD <10ng/ml which is regarded as deficient [32] to be associated with significantly increased risk of total mortality and COPD mortality in those with restrictive and mild/moderate COPD but not in those with severe COPD which was independent of known potential confounders.

Vitamin D deficiency and COPD

It is now established that vitamin D deficiency (variously defined) is highly prevalent in patients with COPD [6,7,8,9,12]. In line with these studies we have shown vitamin D deficiency (<10ng/ml) to be associated with increased risk of having COPD compared to those with sufficient vitamin D levels and the prevalence of vitamin D deficiency increased with increasing severity of COPD even after taking into account a wide range of possible confounders or mediators including smoking, physical inactivity and inflammation (CRP). Whether vitamin D deficiency is a consequence of COPD or whether it plays a role in the development of COPD is still unclear [7]. Despite the biological evidence that vitamin D may improve lung function through its action on regulating inflammation, inducing antimicrobial peptides and/ or its action on muscle [6,35], the potential for reverse causality and confounding remains problematic.

Prospective population studies on vitamin D and lung function decline or incident COPD have yielded inconsistent results, with some reporting positive findings [10,15,20] while others report null findings [11,16]. Small-scale genetic studies on polymorphisms in the vitamin D binding protein and rate of decline in lung function are also inconclusive [9,36]. Although we did not have incident COPD we have shown that vitamin D deficiency (<10 ng/ml) was related to COPD mortality in all men except those with severe COPD; this was not explained by potential confounders and persisted after taking IL-6 into account. Our findings that COPD mortality in those with mild/moderate COPD is significantly increased only in those with vitamin D deficiency (<10ng/ml) is consistent with findings from a randomised control trial (RCT) in subjects with COPD in which a post-hoc analysis was suggestive of reductions in exacerbations in the vitamin D supplementation when subjects had what was regarded as severe vitamin D deficiency (<10ng/mL) only [37].

For peer review only

It is possible that vitamin D does not play a direct role in the progression of COPD but may prevent the development of COPD and COPD deaths through its prevention of respiratory tract infections. Evidence from both observational and RCTs have shown that vitamin D supplementation prevents respiratory tract infections [38] which are a frequent complication of COPD and have been implicated in the onset and progression and exacerbation of chronic lung disease [39]. The lack of association with COPD deaths in those with severe COPD may be due to the advancement of the disease which may not be viable to detect any benefits of vitamin D. Alternately this may be due to lack of power as 25OHD levels in these patients are low and relatively few patients have sufficient 25OHD levels.

Vitamin D and total mortality in COPD subjects

Relatively few studies have examined vitamin D and mortality in subjects with COPD and of those that have, many have reported null results and most have targeted moderate to very severe COPD patients [20,21,23,24]. Two of the population based studies that have examined the association between vitamin D and mortality stratified by lung function status did not show low vitamin D to be independently associated with increased mortality [21,22] . However, in both these studies those with severe COPD were included and vitamin D deficiency (<10 ng/ml) specifically was not examined. Thus the null findings with mortality in previous reports may be due to either the inclusion of those with severe COPD who are at an advanced stage of the disease, in whom vitamin D treatment will have little effect and /or the levels of vitamin D studied. However two recent prospective studies have shown that vitamin D or low vitamin D was associated with all- cause mortality in those with COPD in the general population [25,26]. A recent Cochrane review of vitamin D supplementation trials provided evidence that vitamin D_3 supplementation seem to decrease mortality in elderly adults [40]. The evidence from this

For peer review only

observation study and previous RCT trials in the elderly suggest that vitamin D supplementation in older adults with milder forms of lung disease may particularly benefit from vitamin D supplementation.

Strengths and limitations

This study is based on a cohort of older (60-79 year old) men who constitute a high risk group for vitamin D deficiency and COPD. The study population is socially representative of the UK, and follow-up rates in the British Regional Heart Study are exceptionally high. We were able to take into account a wide range of confounders and inflammatory markers. Our biochemical assays were based on routine clinical assays and are therefore robust. However, blood measurements were based on a single measurement, raising the possibility that the strengths of associations may have been underestimated. In addition, we cannot preclude the possibility of residual confounding. It was based on an older predominantly white male population of European extraction, so that the results cannot be generalized directly to women, younger populations or other ethnic groups. Like most other epidemiological studies we did not have lung function post bronchodilator. Although the present study was a prospective observational study and not a randomised trial, the association between vitamin D deficiency and mortality appeared independent of confounding factors and inflammation.

Conclusion

Our findings suggest that older adults with vitamin D deficiency (<10ng/ml) and earlier stages of lung impairment may potentially benefit from vitamin D supplementation.

For peer review only

Intervention trials in older people with mild or moderate impaired lung function are needed to confirm whether increasing vitamin D levels through supplements in those with vitamin D deficiency will reduce both risk of COPD deaths and overall mortality.

FUNDING SOURCES The British Regional Heart Study is a Research Group supported by the British Heart Foundation (BHF) Programme grant (RG/19/4/34452).

AUTHORS' CONTRIBUTORSHIP STATEMENT

SGW initiated the concept and design of the paper, analysed the data and drafted the manuscript. PW and PHW contributed to the interpretation of data. OP contributed to the analysis of the paper. PW, LL and PHW contributed to the acquisition of the data. All authors revised it critically for important intellectual content and approved the final version of the manuscript.

For peer review only

References

1. Rabe KF, Watz H. Chronic obstructive pulmonary disease. Lancet 2017;389:1931-1940.
2. Holick MF. Vitamin D deficiency . N Eng J Med 2007;357:266-281.
3. Brøndum-Jacobsen P, Benn M, Jensen GB, Nordestgaard BG. 25-hydroxyvitamin d levels and risk of ischemic heart disease, myocardial infarction, and early death: population-based study and meta-analyses of 18 and 17 studies. Arterioscler Thromb Vasc Biol. 2012 Nov;32(11):2794-802.
4. Forouhi NG, Ye Z, Rickard AP, Khaw KT, Luben R, Langenberg C, Wareham NJ. Circulating 25-hydroxyvitamin D concentration and the risk of type 2 diabetes: results from the European Prospective Investigation into Cancer (EPIC)-Norfolk cohort and updated meta-analysis of prospective studies. Diabetologia. 2012;55(8):2173-82.
5. Gandini S, Boniol M, Haukka J, Byrnes G, Cox B, Sneyd MJ, Mullie P, Autier P. Meta-analysis of observational studies of serum 25-hydroxyvitamin D levels and colorectal, breast and prostate cancer and colorectal adenoma. Int J Cancer. 2011;128:1414-24.
6. Ginde AA, Mansbach JM, Camargo CA Jr. Association between serum 25-hydroxyvitamin D level and upper respiratory tract infection in the Third National Health and Nutrition Examination Survey. Arch Intern Med. 2009;169:384-90.
7. Janssens W, Decramer M, Mathieu C, Korf H Vitamin D and chronic obstructive pulmonary disease: hype or reality? Lancet Respir Med. 2013;1(10):804-12.
8. Zhu M, Wang T, Wang C, Ji Y. The association between vitamin D and COPD risk, severity, and exacerbation: an updated systematic review and meta-analysis. Int J COPD 2016:11 2597-2607.
9. Persson LJ, Aanerud M, Hiemstra PS, Hardie JA, Bakke PS, Eagan TM. Chronic obstructive pulmonary disease is associated with low levels of vitamin D. PLoS One. 2012;7(6):e38934.
10. Afzal S, Lange P, Bojesen SE, Freiberg JJ, Nordestgaard BG. Plasma 25-hydroxyvitamin D, lung function and risk of chronic obstructive pulmonary disease. Thorax 2014;69:24-31.

For peer review only

11. Skaaby T, Husemoen LL, Thuesen BH, Pisinger C, Jørgensen T, Fenger RV, Linneberg A. Vitamin D status and chronic obstructive pulmonary disease: a prospective general population study PLoS One. 2014;9(3):e90654.
12. Shaheen SO, Jameson KA, Robinson SM, Boucher BJ, Syddall HE, Sayer AA, Cooper C, Holloway JW, Dennison EM. Relationship of vitamin D status to adult lung function and COPD. Thorax. 2011;66:692-8
13. Wright RJ, Black PN, Scragg R. Relationship between serum 25-hydroxyvitamin D and pulmonary function in the third national health and nutrition examination survey. Chest 2005;128:3792-3798.
14. Choi CJ, Seo M, Choi WS, Kim KS, Youn SA, Lindsey T, Choi YJ, Kim CM. Relationship between serum 25-hydroxyvitamin D and lung function among Korean adults in Korea National Health and Nutrition Examination Survey (KNHANES), 2008-2010. J Clin Endocrinol Metab. 2013;98(4):1703-10.
15. Lange NE, Saprrow D, Vokonas P, Litonjua AA. Vitamin D deficiency, smoking and lung function in the normative aging study. Am J Respir Crit Care Med 2012;186:612-21.
16. Kunisaki KM, Niewoehner DE, Singh RJ, Connett JE. Vitamin D status and longitudinal lung function decline in the Lung Health Study. Eur Respir J. 2011;37(2):238-43.
17. Schöttker B, Haug U, Schomburg L, Köhrle J, Perna L, Müller H, Holleczek B, Brenner H. Strong associations of 25-hydroxyvitamin D concentrations with all-cause, cardiovascular, cancer, and respiratory disease mortality in a large cohort study. Am J Clin Nutr. 2013;97(4):782-93.
18. Khaw KT, Luben R, Wareham N. Serum 25-hydroxyvitamin D, mortality, and incident cardiovascular disease, respiratory disease, cancers, and fractures: a 13-y prospective population study. Am J Clin Nutr. 2014;100(5):1361-70.
19. Chowdhury R, Kunutsor S, Vitezova A, Oliver-Williams C, Chowdhury S, Kiefte-de-Jong JC, Khan H, Baena CP, Prabhakaran D, Hoshen MB, Feldman BS, Pan A, Johnson L, Crowe F, Hu FB, Franco OH. Vitamin D and risk of cause specific death: systematic review and meta-analysis of observational cohort and randomised intervention studies. BMJ. 2014;348:g1903.
20. Persson LJ, Aanerud M, Hiemstra PS, Michelsen AE, Ueland T, Hardie JA, Aukrust P, Bakke PS, Eagan TM. Vitamin D, vitamin D binding protein, and longitudinal outcomes in COPD. PLoS One. 2015;10(3):e0121622.

For peer review only

21. Ford ES. Lung function, 25-hydroxyvitamin D concentrations and **mortality** in US adults. Eur J Clin Nutr. 2015;69:572-8.
22. Lee HM, Liu M, Lee K, Luo Y, Wong ND. Does low vitamin D amplify the association of COPD with total and cardiovascular disease mortality? Clin Cardiol. 2014;37(8):473-8.
23. Holmgaard DB, Mygind LH, Titlestad IL, Madsen H, Fruekilde PB, Pedersen SS, Pedersen C Serum vitamin D in patients with chronic obstructive lung disease does not correlate with mortality--results from a 10-year prospective cohort study.PLoS One. 2013;8(1):e53670.
24. Puhan MA, Siebeling L, Frei A, Zoller M, Bischoff-Ferrari H, ter Riet G. No association of 25-Hydroxyvitamin D with exacerbations in primary care Patients with COPD. Chest 2014;145:37-43.
25. Mattila T, Vasankari T, Rissanen H, Knekt P, Sares-Jaske L,Jaaskelainen T, Heliovaara M. Airway obstruction , serum vitamin D and mortality in a 33 year follow-up study. Eur J Clin Nutr 2019;73:1024-1032.
26. Færk G, Çolak Y, Afzal S, Nordestgaard BG.Færk G, et al. Low concentrations of 25-hydroxyvitamin D and long-term prognosis of COPD: a prospective cohort study. Eur J Epidemiol. 2018 Jun;33(6):567-577
27. Lennon LT, Ramsay SE, Papacosta O, Shaper AG, Wannamethee SG, Whincup PH. Cohort Profile Update: The British Regional Heart Study 1978-2014: 35 years follow-up of cardiovascular disease and ageing. Int J Epidemiol. 2015;44:826-826g.
28. Wannamethee SG, Welsh P, Papacosta O, Lennon L, Whincup PH, Sattar N. Elevated parathyroid hormone, but not vitamin D deficiency, is associated with increased risk of heart failure in older men with and without cardiovascular disease. Circ Heart Fail. 2014;7:732-9.
29. Wannamethee SG, Shaper AG, Papacosta O, Lennon L, Welsh P, Whincup PH Lung function and airway obstruction: associations with circulating markers of cardiac function and incident heart failure in older men-the British Regional Heart Study. Thorax. 2016;71(6):526-34.
30. Knox, S, Harris, J, Calton, L, Wallace, AM. A simple automated solid-phase extraction procedure for measurement of 25-hydroxyvitamin D3 and D2 by liquid chromatography-tandem mass spectrometry. Ann Clin Biochem 2009;46(Pt 3):226–230

For peer review only

31. Bouillon RA, Auwerx JH, Lissens WD, Pelemans WK . Vitamin D status in the elderly: seasonal substrate deficiency causes 1,25-dihydroxycholecalciferol deficiency. Am J Clin Nutr 1987;45:755–763.
32. Ross AC, Manson JE, Abrams SA, Aloia JF, Brannon PM, Clinton SK, Durazo-Arvizu RA, Gallagher JC, Gallo RL, Jones G, Kovacs CS, Mayne ST, Rosen CJ, Shapses SA. The 2011 report on dietary reference intakes for calcium and vitamin D from the Institute of Medicine: what clinicians need to know. J Clin Endocrinol Metab. 2011;96:53-8
33. Falaschetti E, Laiho J, Primatesta P, Purdon S. Prediction equations for normal and low lung function from the health Survey for England. Eur Respir J 2004;456-463.
34. Rabe KF, Hurd S, Anzueto A, Barnes PJ, Buist SA, Calverley P, Fukuchi Y, Jenkins C, Rodriguez-Roisin R, van Weel C, Zielinski J; Global Initiative for Chronic Obstructive Lung Disease. Global strategy for the diagnosis, management, and prevention of chronic obstructive pulmonary disease: GOLD executive summary. Am J Respir Crit Care Med. 2007;176:532-55. Review.
35. Herr C, Greulich T, Koczulla RA, Meyer S, Zakharkina T, Branscheidt M, Eschmann R, Bals R. The role of vitamin D in pulmonary disease: COPD, asthma, infection, and cancer. Respir Res. 2011 Mar 18;12:31.
36. Hansen JG, Gao W, Dupuis J, O'Connor GT, Tang W, Kowgier M, Sood A, Gharib SA, Palmer LJ, Fornage M, Heckbert SR, Psaty BM, Booth SL; SUNLIGHT Consortium., Cassano PA Association of 25-Hydroxyvitamin D status and genetic variation in the vitamin D metabolic pathway with FEV1 in the Framingham Heart Study. Respir Res. 2015;16:81.
37. Lehouck A, Mathieu C, Carremans C, Baeke F, Verhaegen J, Van Eldere J, Decallonne B, Bouillon R, Decramer M, Janssens W. High doses of vitamin D to reduce exacerbations in chronic obstructive pulmonary disease: a randomized trial. Ann Intern Med. 2012;156(2):105-14.
38. Martineau AR, Jolliffe DA, Hooper RL, Greenberg L, Aloia JF, Bergman P, Dubnov-Raz G, Esposito S, Ganmaa D, Ginde AA, Goodall EC, Grant CC, Griffiths CJ, Janssens W, Laaksi I, Manaseki-Holland S, Mauger D, Murdoch DR, Neale R, Rees JR, Simpson S Jr, Stelmach I, Kumar GT, Urashima M, Camargo CA Jr. Vitamin D supplementation to prevent acute respiratory tract infections: systematic review and meta-analysis of individual participant data. BMJ. 2017;356:i6583. doi: 10.1136/bmj.i6583. Review.

For peer review only

39. Sethi S. Infection as a comorbidity of COPD. Eur Respir J. 2010 ;35:1209-15. Review.

40. Bjelakovic G, Gluud LL, Nikolova D, Whitfield K, Wetterslev J, Simonetti RG, Vitamin D supplementation for prevention of mortality in adults. Cochrane Database Syst Rev. 2014;1:CD007470. Review.

For peer review only

Table 1

Table 1 Baseline characteristics by vitamin D levels in men without history of HF

	25OHD (ng/mL)			*p-trend*
	<10 (N=363)	10-19 (N=1499)	≥20 (N=1713)	
Age (yrs)	69.6 (5.8)	68.7 (5.5)	68.4 (5.4)	*0.0003*
%smokers	25.3	12.8	9.5	*<0.0001*
% manual	56.2	52.7	54.9	*0.77*
% inactive	56.4	34.9	27.9	*<0.0001*
% heavy drinkers	6.1	2.9	3.7	*0.01*
%MI/stroke	18.2	13.6	13.4	*0.07*
% diabetes	16.5	14.1	11.0	*<0.0001*
% antihypertensive	38.6	32.0	31.6	*0.04*
% renal dysfunction	15.4	15.6	14.9	*0.63*
BMI (kg/m^2)	26.8 (4.5)	27.1 (3.8)	26.6 (3.3)	*0.007*
% obese	19.6	18.5	13.3	*<0.0001*
SBP (mmHg)	152.4 (26.1)	148.6 (23.9)	149.1 (23.6)	*0.16*
CRP (mg/L)*	2.25	1.65	1.57	*<0.0001*
IL-6	3.0	2.50	2.27	*<0.0001*
**Lung function**				
FEV1 (L)	2.35 (0.71)	2.58 (0.65)	2.67 (0.64)	*<0.0001*
% FEV predicted	80 (23)	86 (21)	89 (21)	*<0.0001*
Abnormal FEV1	12.3	4.9	3.9	*<0.0001*
FVC (L)	3.17 (0.90)	3.36 (0.85)	3.47 (0.83)	*<0.0001*
% FVC predicted	81 (20)	85 (19)	88 (19)	*<0.0001*
Abnormal FVC	9.1	5.5	4.1	*<0.0001*
FEV_1/FVC	0.74 (0.13)	0.77 (0.12)	0.77 (0.11)	*<0.0001*
%COPD	32.5	22.0	21.0	*<0.0001*
mild	6.3	6.5	7.8	*0.16*
Moderate	15.4	11.5	9.6	*0.001*
Severe	10.7	3.9	3.6	<0.0001
% restrictive lung disorder	28.7	30.2	26.5	0.07

Mean and SD unless specified; *Geometric mean and interquartile range

For peer review only

Table 2

Restrictive and obstructive lung function patterns and associations with serum vitamin D and vitamin D deficiency

			Obstructive		
	Normal N=1756	Restrictive N=1011	Mild N=254	Moderate N=394	Severe N=160
**FEV1,% predicted**	101%	75%	95%	66%	39%
**FVC,% predicted**	96%	67%	101%	83%	61%
**25OHD (ng/ml)**					
Mean (SD)	20.71 (0.21)	19.62 (0.29)	20.95 (0.61)	18.86 (0.81)	17.60 (0.74)
Age and season adjusted mean difference	0	-1.05 (-1.76,-0.32)	0.38 (-0.82,1.58)	-1.74 (-2.74,-0.74)	-2.86 (-3.36,-1.36)
Model 1	0	-0.69 (-1.38,0.00)	1.15 (-0.03,2.33)	-0.97 (-1.98,0.06)	-1.48 (-2.80,0.00)
Model 2	0	-0.52 (-1.21,0.20)	1.00 (-0.18,2.18)	-0.85 (-1.85,0.16)	-1.64 9-2.58,0.23)
% vitamin D deficiency	8.0	10.3	9.1	14.2	24.4
Age and season adjusted	1.00	1.36 (1.04,1.78)	0.99 (0.62,1.59)	1.82 (1.30,2.55)	3.52 (2.33,5.30)
Model 1	1.00	1.09 (0.82,1.45)	0.85 (0.51,1.40)	1.49 (1.04,2.14)	2.11 (1.34,3.32)
Model 2	1.00	1.01 (0.75,1.35)	0.90 (0.54,1.48)	1.47 (1.03,2.11)	1.98 (1.26,3.14)

Model 1: adjusted for age, season, smoking, physical activity, social class, BMI, diabetes, renal dysfunction and pre-existing CVD

Model 2=Model 1+IL-6.

For peer review only

Table 3 Vitamin D levels and total, cardiovascular and respiratory mortality

	Vitamin D (ng/ml)				
	<10 (deficient) (N=363)	10-19 (N=1499)	20+ (N=1713)	HR per 1 ng/ml increase in 25OHD	p-linear trend
**Total**					
Rate/1000	67.8 (287)	46.4 (988)	40.9 (1052)		
Age+season	1.80 (1.57,2.05)	1.16 (1.06,1.27)	1.00	0.983 (0.978,0.988)	<0.0001
Model 1	1.52 (1.32,1.75)	1.14 (1.04,1.25)	1.00	0.989 (0.984,0.994)	<0.0001
Model 2	1.47 (1.28,1.70)	1.12 (1.02,1.22)	1.00	0.990 (0.985,0.995)	<0.0001
**CVD**					
Rate/1000	20.6 (87)	15.6 (332)	14.0 (359)		
Model 1	1.28 (0.99,1.65)	1.11 (0.95,1.30)	1.00	0.992 (0.983,1.000)	0.06
Model 2	1.23 (0.95,1.59)	1.07 (0.92,1.26)	1.00	0.993 (0.985,1.002)	0.15
**Respiratory**					
Rate/1000	11.4 (48)	4.8 (103)	4.4 (113)		
Model 1	2.07 (1.44,2.99)	1.08 (0.82,1.42)	1.00	0.978 (0.964,0.993)	0.005
Model 2	2.01 (1.39,2.90)	1.06 (0.80,1.40)	1.00	0.979 (0.964,0.994)	0.007
COPD					
Rate/1000	5.7 (24)	2.3 (48)	1.6 (42)		
Model 1	2.25 (1.31,3.88)	1.43 (0.93,2.19)	1.00	0.972 (0.950,0.994)	0.01
Model 2	2.06 (1.19,3.58)	1.37 (0.88,2.11)	1.00	0.975 (0.953,0.997)	0.02

Model 1: adjusted for age, season, smoking, physical activity, social class, BMI, diabetes, use of antihypertensive treatment, pre-existing CVD and impaired lung function.

Model 2=Model 1+IL-6.

For peer review only

Table 4 Vitamin D levels and total mortality by patterns of lung function

	25(OH)D (ng/ml)			
	<10 (deficient)	10-19	20+	*p-linear trend*
**None (N=1756)**				
Rate/1000 (n/N)	52.9 (100/141)	35.7 (397/716)	34.4 (490/899)	
Model 1	1.45 (1.15,1.82)	1.06 (0.92,1.22)	1.00	*0.02*
Model 2	1.39 (1.10,1.75)	1.05 (0.91,1.22)	1.00	*0.04*
**Restrictive (N=1011)**				
Rate/1000 (n/N)	74.4 (86/104)	51.8 (341/453)	41.6 (305/454)	
Model 1	1.52 (1.19,2.00)	1.27 (1.08,1.50)	1.00	*0.002*
Model 2	1.52 (1.17,1.98)	1.26 (1.07,1.49)	1.00	*0.002*
**Mild/Moderate COPD (N=648)**				
Rate/1000 (n/N)	75.3 (65/79)	56.7 (199/271)	49.0 (207/298)	
Model 1	1.68 (1.25,2.25)	1.18 (0.96,1.45)	1.00	*0.02*
Model 2	1.58 (1.17,2.14)	1.11 (0.90,1.36)	1.00	*0.14*
**Severe COPD (N=160)**				
Rate/1000 (n/N)	96.4 (36/39)	71.8 (51/59)	65.0 (50/62)	
Model 1	1.43 (0.85,2.38)	0.87 (0.57,1.34)	1.00	*0.56*
**Model 2**	1.39 (0.83,2.33)	0.85 (0.55,1.30)	1.00	*0.64*

Model 1: adjusted for age, season, smoking, physical activity, social class, BMI, diabetes, use of antihypertensive treatment and pre-existing CVD

Model 2=Model 1+IL-6.

For peer review only

Table 5 Vitamin D and respiratory and COPD deaths by patterns of lung function

	25(OH)D (ng/ml)		
	<10 (deficient)	10-19	20+
**RESPIRATORY deaths**			
**None**			
Rates/1000 p-yrs	4.2 (8/141)	2.6 (29/716)	2.8 (40/899)
Model 1	1.63 (0.74,3.62)	0.99 (0.60,1.62	1.00
**Restrictive**			
Rates/1000 p-yrs	11.6 (13/104)	5.8 (35/453)	4.9 (32/454)
Model 1	2.34 (1.16,4.71)	1.26 (0.75,2.10)	1.00
**Mild/Moderate COPD**			
Rates/1000 p-yrs	15.0 (13/79)	6.8 (24/271)	6.2 (26/298)
Model 1	2.91 (1.42,5.97)	1.16 (0.65,2.08)	1.00
**Severe COPD**			
Rates/1000 p-yrs	23.3 (14/39)	20.5 (15/59)	20.5 (15/62)
Model 1	1.27 (0.53,3.03)	0.65 0.28,1.50)	1.00
**COPD deaths**			
**None**			
Rates/1000 p-yrs	1.0 (2/141)	0.4 (4/716)	0.4 (6/899)
Model 1	4.78 (0.86,26.70)	1.13 (0.30,4.29)	1.00
**Restrictive**			
Rates/1000 p-yrs	2.7 (3/104)	2.8 (17/453)	0.9 (6/454)
Model 1	3.26 (0.72,14.80)	3.64 (1.36,9.71)	1.00
**Mild/Moderate COPD**			
Rates/1000 p-yrs	11.4 (9/79)	4.9 (17/271)	3.9 (16/298)
Model 1	3.75 (1.55,9.12)	1.46 (0.71,3.00)	1.00
**Severe COPD**			
Rates/1000 p-yrs	28.2 (10/39)	15.5 (10/59)	19.1 (14/62)
Model 1	1.04 (0.40,2.73)	0.47 (0.18,1.26)	1.00

Model 1: adjusted for age, season, smoking, physical activity, social class, BMI, diabetes, use of antihypertensive treatment and pre-existing CVD

For peer review only

STROBE Statement—checklist of items that should be included in reports of observational studies

	Item No	Recommendation	Page No
**Title and abstract**	1	(*a*) Indicate the study's design with a commonly used term in the title or the abstract	2
		(*b*) Provide in the abstract an informative and balanced summary of what was done and what was found	2
**Introduction**			
Background/rationale	2	Explain the scientific background and rationale for the investigation being reported	5
Objectives	3	State specific objectives, including any prespecified hypotheses	6
**Methods**			
Study design	4	Present key elements of study design early in the paper	6
Setting	5	Describe the setting, locations, and relevant dates, including periods of recruitment, exposure, follow-up, and data collection	6-7
Participants	6	(*a*) *Cohort study*—Give the eligibility criteria, and the sources and methods of selection of participants. Describe methods of follow-up<br>*Case-control study*—Give the eligibility criteria, and the sources and methods of case ascertainment and control selection. Give the rationale for the choice of cases and controls<br>*Cross-sectional study*—Give the eligibility criteria, and the sources and methods of selection of participants	6-8
		(*b*) *Cohort study*—For matched studies, give matching criteria and number of exposed and unexposed<br>*Case-control study*—For matched studies, give matching criteria and the number of controls per case	
Variables	7	Clearly define all outcomes, exposures, predictors, potential confounders, and effect modifiers. Give diagnostic criteria, if applicable	7-8
Data sources/ measurement	8*	For each variable of interest, give sources of data and details of methods of assessment (measurement). Describe comparability of assessment methods if there is more than one group	*7-8*
Bias	9	Describe any efforts to address potential sources of bias	14
Study size	10	Explain how the study size was arrived at	
Quantitative variables	11	Explain how quantitative variables were handled in the analyses. If applicable, describe which groupings were chosen and why	8
Statistical methods	12	(*a*) Describe all statistical methods, including those used to control for confounding	8
		(*b*) Describe any methods used to examine subgroups and interactions	8
		(*c*) Explain how missing data were addressed	8
		(*d*) *Cohort study*—If applicable, explain how loss to follow-up was addressed<br>*Case-control study*—If applicable, explain how matching of cases and controls was addressed<br>*Cross-sectional study*—If applicable, describe analytical methods taking account of sampling strategy	
		(*e*) Describe any sensitivity analyses	

Continued on next page

For peer review only - http://bmjopen.bmj.com/site/about/guidelines.xhtml

**Results**			
Participants	13*	(a) Report numbers of individuals at each stage of study—eg numbers potentially eligible, examined for eligibility, confirmed eligible, included in the study, completing follow-up, and analysed	6
		(b) Give reasons for non-participation at each stage	6
		(c) Consider use of a flow diagram	
Descriptive data	14*	(a) Give characteristics of study participants (eg demographic, clinical, social) and information on exposures and potential confounders	6-8
		(b) Indicate number of participants with missing data for each variable of interest	6
		(c) *Cohort study*—Summarise follow-up time (eg, average and total amount)	8
Outcome data	15*	*Cohort study*—Report numbers of outcome events or summary measures over time	*9*
		Case-control study—Report numbers in each exposure category, or summary measures of exposure	
		Cross-sectional study—Report numbers of outcome events or summary measures	
Main results	16	(*a*) Give unadjusted estimates and, if applicable, confounder-adjusted estimates and their precision (eg, 95% confidence interval). Make clear which confounders were adjusted for and why they were included	10-11
		(*b*) Report category boundaries when continuous variables were categorized	9
		(*c*) If relevant, consider translating estimates of relative risk into absolute risk for a meaningful time period	
Other analyses	17	Report other analyses done—eg analyses of subgroups and interactions, and sensitivity analyses	11
**Discussion**			
Key results	18	Summarise key results with reference to study objectives	11
Limitations	19	Discuss limitations of the study, taking into account sources of potential bias or imprecision. Discuss both direction and magnitude of any potential bias	14
Interpretation	20	Give a cautious overall interpretation of results considering objectives, limitations, multiplicity of analyses, results from similar studies, and other relevant evidence	11-14
Generalisability	21	Discuss the generalisability (external validity) of the study results	14
**Other information**			
Funding	22	Give the source of funding and the role of the funders for the present study and, if applicable, for the original study on which the present article is based	15

*Give information separately for cases and controls in case-control studies and, if applicable, for exposed and unexposed groups in cohort and cross-sectional studies.

Note: An Explanation and Elaboration article discusses each checklist item and gives methodological background and published examples of transparent reporting. The STROBE checklist is best used in conjunction with this article (freely available on the Web sites of PLoS Medicine at http://www.plosmedicine.org/, Annals of Internal Medicine at http://www.annals.org/, and Epidemiology at http://www.epidem.com/). Information on the STROBE Initiative is available at www.strobe-statement.org.

For peer review only

BMJ Open

Vitamin D deficiency, impaired lung function and total and respiratory mortality in a cohort of older men: Cross sectional and prospective findings from The British Regional Heart Study

Journal:	*BMJ Open*
Manuscript ID	bmjopen-2021-051560.R1
Article Type:	Original research
Date Submitted by the Author:	26-Jul-2021

Complete List of Authors:	Wannamethee, Goya; UCL Medical School, UCL Department of Primary Care & Population Health<br>Welsh, Paul; University of Glasgow, Institute of Cardiovascular and Medical Sciences<br>Papacosta, Olia; UCL Medical School, Primary Care and Population Health<br>Lennon, Lucy; University College London Medical School, Primary Care and Population Health<br>Whincup, Peter; St George's University of London

**Primary Subject Heading**:	Epidemiology
Secondary Subject Heading:	Respiratory medicine, Nutrition and metabolism
Keywords:	EPIDEMIOLOGY, RESPIRATORY MEDICINE (see Thoracic Medicine), NUTRITION & DIETETICS

SCHOLARONE™
Manuscripts

I, the Submitting Author has the right to grant and does grant on behalf of all authors of the Work (as defined in the below author licence), an exclusive licence and/or a non-exclusive licence for contributions from authors who are: i) UK Crown employees; ii) where BMJ has agreed a CC-BY licence shall apply, and/or iii) in accordance with the terms applicable for US Federal Government officers or employees acting as part of their official duties; on a worldwide, perpetual, irrevocable, royalty-free basis to BMJ Publishing Group Ltd ("BMJ") its licensees and where the relevant Journal is co-owned by BMJ to the co-owners of the Journal, to publish the Work in this journal and any other BMJ products and to exploit all rights, as set out in our licence.

The Submitting Author accepts and understands that any supply made under these terms is made by BMJ to the Submitting Author unless you are acting as an employee on behalf of your employer or a postgraduate student of an affiliated institution which is paying any applicable article publishing charge ("APC") for Open Access articles. Where the Submitting Author wishes to make the Work available on an Open Access basis (and intends to pay the relevant APC), the terms of reuse of such Open Access shall be governed by a Creative Commons licence – details of these licences and which Creative Commons licence will apply to this Work are set out in our licence referred to above.

Other than as permitted in any relevant BMJ Author's Self Archiving Policies, I confirm this Work has not been accepted for publication elsewhere, is not being considered for publication elsewhere and does not duplicate material already published. I confirm all authors consent to publication of this Work and authorise the granting of this licence.

For peer review only

Vitamin D deficiency, impaired lung function and total and respiratory mortality in a cohort of older men: Cross sectional and prospective findings from The British Regional Heart Study

S Goya Wannamethee[1], Paul Welsh[2], Olia Papacosta[1], Lucy Lennon[1], Peter H Whincup[3]

1.UCL Department of Primary Care & Population Health, UCL Medical School, Rowland Hill Street, London. NW3 2PF UK

2. Institute of Cardiovascular & Medical Sciences, BHF Glasgow Cardiovascular Research Centre, University of Glasgow

3. Population Health Research Institute, St George's, University of London, Cranmer Terrace, London. SW17 0RE, UK

Address for Correspondence Prof S Goya Wannamethee, Department Primary Care and Population Health, UCL Medical School, Royal Free Campus, Rowland Hill St, London NW32PF, UK.

e-mail g.wannamethee@ucl.ac.uk

Word count text: 3881

Tables 5

Supplementary Table 1

CONFLICT OF INTEREST NONE

For peer review only

ABSTRACT

Objectives Vitamin D deficiency is associated with chronic obstructive pulmonary disease (COPD). We examined the cross-sectional association between 25-hydroxyvitamin D [25(OH)D] and lung function impairment and assessed whether vitamin D deficiency is related to long term mortality in those with impaired lung function.

Design Prospective study

Setting General practices in the UK.

Participants 3575 men aged 60-79 years with no prevalent heart failure.

Outcome measures Airway obstruction and mortality. The Global Initiative on Obstructive Lung diseases (GOLD) spirometry criteria was used to define airway obstruction.

Results During the follow-up period of 20 years, there were 2327 deaths (114 COPD deaths). Vitamin D deficiency was defined as serum 25(OH)D levels <10 ng/ml; insufficiency as 25(OH)D 10-19 ng/mL; sufficient as 25(OH)D ≥20 ng/mL .In cross sectional analysis vitamin D deficiency was more prevalent in those with moderate COPD (FEV/FVC <70% and FEV1 50-80%) and severe COPD (FEV/FVC <70% and FEV1 < 50%) but not in those with mild COPD (FEV/FVC <70% and FEV1 > 80%) or restrictive lung disease (FEV1/FVC >70% and FVC <80%). However, vitamin D deficiency was associated with increased risk of total and respiratory mortality in both men with COPD and men with restrictive lung disease after adjustment for confounders and inflammation. The adjusted hazard ratios (HRs) (95% CI) for total mortality comparing levels of 25(OH)D <10ng/mL to 25(OH)D >20 ng/ml were 1.39 (1.10,1.75), 1.52 (1.17,1.98), 1.58 (1.17,2.14) and 1.39 (0.83,2.33) for those with no lung impairment, restrictive lung function, mild/moderate COPD and severe COPD respectively.

For peer review only

Conclusion   Men with COPD were more likely to be vitamin D deficient than those with normal lung function. Vitamin D deficiency is associated with increased all-cause mortality in older adults with no lung impairment as well as in those with restrictive or obstructive lung impairment.

Key words: Vitamin D deficiency, 25(OH)D; chronic obstructive lung disease; lung function ; mortality

For peer review only

Strengths and limitations of this study

- This study is carried out in a high-risk group for vitamin D deficiency and COPD and examined restrictive and obstructive lung disease separately.

- The study population is socially representative of the UK.

- The study took into account a wide range of confounders and inflammatory markers that could explain the associations between vitamin D deficiency and lung function and mortality.

-. The study is limited by the use of a single measurement of vitamin D [25(OH)D].

- The study is restricted to a predominantly white male population of European extraction,

For peer review only

INTRODUCTION

Chronic obstructive lung disease (COPD) is a common chronic inflammatory disease in the elderly and is a major cause of morbidity and mortality [1]. Much attention has focussed on identifying factors which may influence prognosis and mortality in these patients. Vitamin D deficiency (ascertained from measuring the circulating 25-dehydroxyvitamin D [25(OH)D] metabolite) is recognised as an important health problem particularly in the elderly [2] and has been associated with various diseases including respiratory infections, autoimmune disease, CVD, diabetes and cancer [3-6]. Although vitamin D deficiency is traditionally known for its role in bone health, there is growing interest in the role of vitamin D in the pathogenesis and severity of COPD [7]. Observational studies have found Vitamin D deficiency to be highly prevalent in patients with COPD [8-12]. While many cross-sectional population studies have associated higher 25(OH)D with better lung function as measured by the FEV1 [10-16], prospective studies of the association of baseline 25(OH)D with lung function decline and incident COPD have produced conflicting results [9-11,15,16]. Whether the association between 25(OH)D and impaired lung function is specific to COPD is also unclear. Vitamin D deficiency has been associated with increased mortality in the general population [17-19] but the associations with pulmonary death and total mortality in COPD patients remains uncertain. Longitudinal studies that have examined the association between 25(OH)D and mortality in COPD patients have mostly reported no significant association with mortality [20-24]. However, most of these previous studies have targeted moderate and severe COPD patients only. Two recent prospective studies showed that vitamin D deficiency or low vitamin D was associated with all-cause mortality in those with COPD in the general population [25,26] . Few studies have examined the association between circulating vitamin D deficiency and impaired lung function in the general older population specifically and whether vitamin D deficiency is associated with mortality in older adults

For peer review only

with milder degrees of impaired lung function (restrictive or obstructive) has not been well studied. The aim of the present study is to 1) investigate the association between vitamin D deficiency and both restrictive and obstructive lung function impairment and 2) whether vitamin D deficiency is related to long term mortality in those with impaired lung function.

SUBJECTS AND METHODS

The British Regional Heart Study is a prospective study involving 7735 men aged 40-59 years drawn from one general practice in each of 24 British towns, who were screened between 1978 and 1980 [27]. The population studied was socio-economically representative of British men and comprises predominantly white Europeans (>99%). In 1998-2000, all surviving men, then aged 60-79 years, were invited for a 20th year follow-up examination. All men provided informed written consent. Ethical approval was obtained from the National Research Ethics Service Committee London. All men completed a mailed questionnaire providing information on their lifestyle and medical history, had a physical examination and provided a fasting blood sample. The samples were frozen and stored at -20^{0}C on the day of collection and transferred in batches for storage at -70°C until analysis, carried out after no more than one freeze-thaw cycle. The men were asked whether a doctor had ever told them that they had angina or myocardial infarction (MI), heart failure (HF) or stroke; details of their medications were recorded at the examination including use of bronchodilators (BNF code 3.1). 4252 men (77% of available survivors) attended for examination.

Study subjects

Blood measurements of 25(OH)D, were available in 3754 men (88%) at the 20-year follow-up examination (1998-2000). Of these men, we excluded 88 men with a history of a diagnosis of

For peer review only

HF as these men have exceptional mortality rates and reduced lung function and a further 91 men with no lung function measures leaving 3575 men for analysis.

Cardiovascular risk factor measurements at 1998-2000

Anthropometric measurements including body weight, height, and waist circumference (WC) were carried out. Details of measurement and classification methods for smoking status, physical activity, social class, alcohol intake, blood pressure and blood lipids in this cohort have been described [28,29]. Heavy drinking was defined as drinking more than five units (1 UK unit = 10g) of alcohol daily on most days. Physical activity scores were assigned on the basis of frequency and type of activity and the men were divided into 6 groups: none, occasional, light, moderate, moderately vigorous and vigorous. Subjects who reported none or occasional activity were classified as 'inactive'. On the basis of their reported cigarette smoking status the men were classified as never smokers, long term ex-smokers ($\geq$ 15 years) , recent ex-smokers (<15 years) and current smokers. Social class was derived from the longest held occupation recorded at the time of baseline questionnaire (1978-80) using the Registrar General's classification of occupations, with categories grouped as non-manual (I, II and III non-manual) and manual (III manual, IV and V). Interleukin-6 was assayed using a high-sensitivity ELISA (R & D Systems, Oxford, UK). C-reactive protein (CRP) was assayed by ultra-sensitive nephelometry (Dade Behring, Milton Keynes, UK).

Vitamin D

Measurement of 25(OH)D was performed on EDTA-anticoagulated plasma via a high-throughput method for the measurement of 25(OH)D_3 and -D_2 using a gold-standard liquid chromatography-tandem mass spectrometry method following an automated solid-phase extraction procedure [30,31]. Our method is calibrated and controlled using reagents from

Chromsystems GmbH (Manchester, UK) and is currently in routine clinical use. Results are reported as total 25(OH)D [$25OHD_2$ + $25OHD_3$); virtually all participants had an undetectable $25OHD_2$, which is commensurate with results observed in routine NHS use. The lower limit of sensitivity was 4ng/ml for both $25(OH)D_3$ and total 25(OH)D. Vitamin D deficiency was defined as 25(OH)D <10 ng/ml] and vitamin D insufficiency as 25(OH)D 10-19 ng/ml [2,31,32].

Lung function

Forced expiratory volume in 1 second (FEV1) and forced vital capacity (FVC) were measured using a Vitalograph Compact spirometer with the subject seated [29]. We used predictive equations for FEV1 and FVC that were derived from the general population Health Survey for England for males aged >25 years [33]. The following equations were used:

$$\text{predicted FEV1}=-\exp[-9.37674+0.000183*\text{age}-0.00011*\text{age}^2+\log(\text{height})]$$

$$\text{predicted FVC}= \exp[-10.36706+0.00434*\text{age}-0.00011*\text{age}^2+\log(\text{height})]$$

Predicted FEV1%=raw FEV1/predicted FEV1 and predicted FVC%=raw FVC/predicted FVC. We divided the men into 5 lung pattern groups according to their FEV1/FVC ratio using a modification of the criteria developed by The Global Initiative for Chronic Obstructive Lung Disease (GOLD) for defining the severity stages of obstructive airways [34]: 1) severe airflow obstruction (FEV1/FVC <0.70 and FEV1 <50% predicted), moderate airflow obstruction (FEV1/FVC <0.70 and FEV1 $\geq$ 50 to <80%), mild airflow obstruction (FEV1/FVC <0.70 and FEV1$\geq$ 80%), restricted (FEV1/FVC >0.70 and FVC <80% predicted) and normal (FEV1/FVC >0.70 and FVC >80% and not on bronchodilators).

Follow-up

For peer review only

All men have been followed up from initial examination (1978-1980) for cardiovascular morbidity [27] and follow-up has been achieved for 99% of the cohort. In the present analyses, all-cause mortality is based on follow-up from re-examination in 1998-2000 at mean age 60-79 years to September 2019. Details on causes of deaths were collected through the National Health Service Central Register. COPD deaths were defined as deaths due to chronic bronchitis (ICD-9 codes 490-491), emphysema (ICD-9 code 492), and chronic airway obstruction (ICD-9 code 496).

For peer review only

Statistical analysis

The men were classified into 3 vitamin D groups: 25(OH)D <10ng/ml (deficient), 10-19 ng/ml (insufficient) and ≥ 20 ng/ml (sufficient). Cox's proportional hazards model was used to assess the multivariate-adjusted hazards ratio (relative risk) in a comparison of three vitamin D groups: <10ng/ml, 10-19 ng/ml and ≥20ng/ml and as well as in a 1 unit increase in 25OHD.
All analyses were initially adjusted for age and season. In the multivariate analyses we adjusted further for potential confounders known to be associated with lung function and mortality which included smoking, social class, physical activity, heavy drinking, BMI use of antihypertensive treatment, diabetes and pre-existing CVD. We also carried out supplementary analysis without inclusion of BMI since BMI may be a confounder or a potential mediator. We further adjusted for IL-6 as a potential mediator. In multivariate analyses, smoking, social class, physical activity, heavy drinking, diabetes, use of antihypertensive treatment and pre-existing CVD were fitted as categorical variables; IL-6 and BMI were fitted as fitted as a continuous variable. Analysis of variance was used to obtain adjusted mean differences in 25(OH)D between the lung impairment groups. Logistic regression was used to assess the relative odds

of having vitamin D deficiency (yes/no) for the lung impairment groups compared to those with normal lung function.

Patients and public involvement

Patients and the public were not involved in the conduct, design, analysis and interpretation of this research.

RESULTS

During the mean follow-up period of 20 years there were 2327 deaths from all causes in the 3575 men with no diagnosed HF, including 114 deaths due to COPD. The mean 25(OH)D was higher in men examined in winter (Dec-February) than in those examined in summer (June-- August) 22.2 (9.4) vs 17.1 (7.4) ng/ml. The prevalence of COPD was also much higher in winter than in summer (17% vs 30.2%).

Baseline characteristics

Table 1 summarises baseline characteristics by the three 25(OH)D groups. At baseline, men with vitamin D deficiency had a more adverse health profile including the highest prevalence of smoking, heavy drinking, physical inactivity, obesity, hypertension and the highest mean CRP and IL-6 (markers of inflammation) (Table 1). 25.3% of those with vitamin D deficiency were smokers compared with only 9.5% in those who had vitamin D sufficiency. Lung function (both FEV and FVC) was higher in those with higher 25(OH)D levels. Men with vitamin D deficiency had the highest prevalence of COPD and this was seen for moderate and severe COPD but not for mild COPD. There was no association between vitamin D deficiency and prevalence of restrictive lung impairment. We divided the vitamin

For peer review only

D sufficient group [25(OH)D ≥20 ng/ml] further into those with ranges 20-29, 30-39 and those with ≥40 ng/ml. There was no further increase in lung function with increasing levels of 25(OH)D among the sufficient group (Supplementary Table 1).

Cross sectional associations between restrictive and obstructive lung function patterns and 25(OH)D and vitamin D deficiency

Table 2 shows the absolute mean 25(OH)D for the 5 lung function groups and the adjusted difference in mean 25(OH)D for those with impaired lung function compared to those with normal lung function. Mean 25(OH)D was lower in men with moderate or severe airway obstruction but not in those with mild airway obstruction compared to those with normal function (Table 2). Men with moderate and severe COPD but not those with restrictive lung patterns were more likely to have vitamin D deficiency. Men with moderate and severe COPD showed increased odds of having vitamin D deficiency compared to those with normal lung function even after adjustment for potential confounders including age, season, smoking, physical activity, social class, heavy drinking, BMI, diabetes, and pre-existing CVD (Table 2). These associations persisted after further adjustment for IL-6.

Prospective associations between 25(OHD)2 and Total mortality

25(OH)D was inversely associated with total mortality even after adjustment for potential confounders, BMI and inflammation (IL-6) (Table 3). The inverse association persisted upon exclusion of current smokers. Compared to those with vitamin D sufficiency the adjusted HR (95%CI) were 1.41 (1.19,1.68) and 1.11 (1.00,1.24)] for those with vitamin D deficiency and insufficiency respectively (p=0.002 for trend). The inverse association was seen for mortality

For peer review only

from respiratory and COPD causes but not from CVD causes after adjustment for potential confounders, BMI and inflammation (Table 3).

When examined by lung function status, vitamin D deficiency was associated with increased mortality in those with normal lung function as well as in those with restrictive lung disorder or mild/moderate COPD even after adjustment for confounders and inflammation (Table 4). In men with severe COPD a statistically non- significant increase in mortality was seen possibly due to small numbers. Vitamin D deficiency was associated with increased risk of respiratory mortality even after adjustment although this was statistically significant only in the restrictive and mild and moderate COPD group possibly because of small numbers (Table 5). The number of COPD deaths was small, but vitamin D deficiency was associated with increased risk of COPD mortality in all groups with the exception of those with severe COPD.

Discussion

Our findings confirm previous observational population studies on the association between low 25(OH)D and COPD and total mortality [13-19] and extend these findings further. The association was not explained by smoking, physical inactivity, or inflammation (IL-6) which are known to relate to both COPD and vitamin D deficiency [35]. Restrictive lung function, by contrast, was not associated with vitamin D deficiency. The definition of vitamin D deficiency and insufficiency remains controversial; the United Kingdom's National Institute for Health and Care Excellence defines it as <25 nmol/l (<10 ng/ml) [36], while the United States' Institute of Medicine defines it as <30 nmol/l (<12.5 ng/ml) [37]. We have shown that 25(OH)D <10ng/ml which is regarded as deficient [32] to be associated with increased risk of

For peer review only

total mortality and COPD mortality in those with restrictive and mild/moderate COPD but not in those with severe COPD which was independent of known potential confounders.

Levels of 50 nmol/L (20 ng/mL) or more are sufficient for most people [37]. In contrast, the Endocrine Society stated that, for clinical practice, a serum 25(OH)D concentration of more than 75 nmol/L (30 ng/mL) is necessary to maximize the effect of vitamin D on calcium, bone, and muscle metabolism [38]. When those with 25(OH)D concentration >20 ng/ml were divided further there was no evidence that those with levels 20-29 ng/ml had lower lung function or higher mortality than those with levels >30 ng/ml. There has been suggestion that mortality is also increased in those with higher extreme 25(OH)D levels (>100 nmol/l or 40ng/ml) [39]. The number of older men with levels ≥ 40 ng/ml in this study was small and we observed no excess mortality in this group in contrast to the Copenhagen Vitamin D Study where the average age (50 years) was younger [39].

Vitamin D deficiency and COPD

It is now established that vitamin D deficiency (variously defined) is highly prevalent in patients with COPD [6,7,8,9,12]. In line with these studies, we have shown in cross sectional analysis that those with COPD were more likely to have vitamin D deficiency (<10ng/ml) than those with normal lung function and the prevalence of vitamin D deficiency increased with increasing severity of COPD even after taking into account a wide range of possible confounders and mediators including smoking, physical inactivity, BMI, and inflammation (IL-6). Whether vitamin D deficiency is a consequence of COPD or whether it plays a role in the development of COPD is still unclear [7]. Despite the biological evidence that vitamin D may improve lung function through its action on regulating inflammation, inducing antimicrobial peptides and/ or

For peer review only

its action on muscle [6,35], the potential for reverse causality and confounding remains problematic.

Prospective population studies on vitamin D [25(OH)D)]and lung function decline or incident COPD have yielded inconsistent results, with some reporting positive findings [10,15,20] while others report null findings [11,16]. Small-scale genetic studies on polymorphisms in the vitamin D binding protein and rate of decline in lung function are also inconclusive [9,40]. Although we did not have incident COPD, we have shown that vitamin D deficiency (25OHD <10 ng/ml) was related to COPD mortality in all men except those with severe COPD; this was not explained by potential confounders and persisted after taking IL-6 into account. Our findings that COPD mortality in those with mild/moderate COPD is significantly increased only in those with vitamin D deficiency is consistent with findings from a randomised control trial (RCT) in subjects with COPD in which a post-hoc analysis was suggestive of reductions in exacerbations in the vitamin D supplementation when subjects had what was regarded as severe vitamin D deficiency (25OHD <10ng/mL) only [41].

It is possible that vitamin D does not play a direct role in the progression of COPD but may prevent the development of COPD and COPD deaths through its prevention of respiratory tract infections. Evidence from both observational and RCTs have shown that vitamin D supplementation prevents respiratory tract infections [42] which are a frequent complication of COPD and have been implicated in the onset and progression and exacerbation of chronic lung disease [43]. The lack of association with COPD deaths in those with severe COPD may be due to the advancement of the disease which may not be viable to detect any benefits of vitamin D.

For peer review only

Alternately this may be due to lack of power as 25(OH)D levels in these patients are low and relatively few patients have sufficient 25(OH)D levels.

Vitamin D and total mortality in COPD subjects

Relatively few studies have examined 25(OH)D (vitamin D) and mortality in subjects with COPD and of those that have, many have reported null results and most have targeted moderate to very severe COPD patients [20,21,23,24]. Two of the population- based studies that have examined the association between 25(OH)D and mortality stratified by lung function status did not show low 25(OH)D (variously defined) to be independently associated with increased mortality [21,22]. However, in both these studies those with severe COPD were included and vitamin D deficiency [25(OH)D <10 ng/ml] specifically was not examined. Thus, the null findings with mortality in previous reports may be due to either the inclusion of those with severe COPD who are at an advanced stage of the disease, in whom vitamin D treatment will have little effect and /or the levels of 25(OH)D studied. However, two recent prospective studies have shown that 25(OH)D or low 25(OH)D (defined as <12.5 nmol/l) was associated with all- cause mortality in those with COPD in the general population [25,26]. A recent Cochrane review of vitamin D supplementation trials provided evidence that vitamin D_3 supplementation seem to decrease mortality in elderly adults [44].

Strengths and limitations

This study is based on a cohort of older (60–79-year-old) men who constitute a high-risk group for vitamin D deficiency and COPD. The study population is socially representative of the UK, and follow-up rates in the British Regional Heart Study are exceptionally high.

For peer review only

We were able to take into account a wide range of confounders and inflammatory markers. Our biochemical assays were based on routine clinical assays and are therefore robust. However, blood measurements were based on a single measurement, raising the possibility that the strengths of associations may have been underestimated. In addition, we cannot preclude the possibility of residual confounding. It was based on an older predominantly white male population of European extraction, so that the results cannot be generalized directly to women, younger populations, or other ethnic groups. Like most other epidemiological studies, we did not have lung function post bronchodilator. Although the present study was a prospective observational study and not a randomised trial, the association between vitamin D deficiency and mortality appeared independent of confounding factors and inflammation.

Conclusion

Men with COPD but not men with restrictive lung function were more likely to be vitamin D deficient than those with normal lung function. Vitamin D deficiency was associated with increased total mortality and mortality from respiratory and COPD causes in older adults, and this was seen in those without lung impairment and in those with earlier stages of lung impairment. Intervention trials in older people with mild or moderate impaired lung function are needed to confirm whether increasing vitamin D through supplements in those with vitamin D deficiency will reduce both risk of COPD deaths and overall mortality.

DATA SHARING STATEMENT

Data are available upon reasonable request.

For peer review only

The deidentified participant data that support the findings of this study are available from the corresponding author upon reasonable request (contact Lucy Lennon at l.lennon@ucl.ac.uk).

FUNDING SOURCES The British Regional Heart Study is a Research Group supported by the British Heart Foundation (BHF) Programme grant (RG/19/4/34452).

AUTHORS' CONTRIBUTORSHIP STATEMENT

SGW initiated the concept and design of the paper, analysed the data and drafted the manuscript. PW and PHW contributed to the interpretation of data. OP contributed to the analysis of the paper. PW, LL and PHW contributed to the acquisition of the data. All authors revised it critically for important intellectual content and approved the final version of the manuscript.

For peer review only

References

1. Rabe KF, Watz H. Chronic obstructive pulmonary disease. Lancet 2017;389:1931-1940.
2. Holick MF. Vitamin D deficiency . N Eng J Med 2007;357:266-281.
3. Brøndum-Jacobsen P, Benn M, Jensen GB, Nordestgaard BG. 25-hydroxyvitamin d levels and risk of ischemic heart disease, myocardial infarction, and early death: population-based study and meta-analyses of 18 and 17 studies. Arterioscler Thromb Vasc Biol. 2012 Nov;32(11):2794-802.
4. Forouhi NG, Ye Z, Rickard AP, Khaw KT, Luben R, Langenberg C, Wareham NJ. Circulating 25-hydroxyvitamin D concentration and the risk of type 2 diabetes: results from the European Prospective Investigation into Cancer (EPIC)-Norfolk cohort and updated meta-analysis of prospective studies. Diabetologia. 2012;55(8):2173-82.
5. Gandini S, Boniol M, Haukka J, Byrnes G, Cox B, Sneyd MJ, Mullie P, Autier P. Meta-analysis of observational studies of serum 25-hydroxyvitamin D levels and colorectal, breast and prostate cancer and colorectal adenoma. Int J Cancer. 2011;128:1414-24.
6. Ginde AA, Mansbach JM, Camargo CA Jr. Association between serum 25-hydroxyvitamin D level and upper respiratory tract infection in the Third National Health and Nutrition Examination Survey. Arch Intern Med. 2009;169:384-90.
7. Janssens W, Decramer M, Mathieu C, Korf H Vitamin D and chronic obstructive pulmonary disease: hype or reality? Lancet Respir Med. 2013;1(10):804-12.
8. Zhu M, Wang T, Wang C, Ji Y. The association between vitamin D and COPD risk, severity, and exacerbation: an updated systematic review and meta-analysis. Int J COPD 2016:11 2597-2607.
9. Persson LJ, Aanerud M, Hiemstra PS, Hardie JA, Bakke PS, Eagan TM. Chronic obstructive pulmonary disease is associated with low levels of vitamin D. PLoS One. 2012;7(6):e38934.
10. Afzal S, Lange P, Bojesen SE, Freiberg JJ, Nordestgaard BG. Plasma 25-hydroxyvitamin D, lung function and risk of chronic obstructive pulmonary disease. Thorax 2014;69:24-31.

For peer review only

11. Skaaby T, Husemoen LL, Thuesen BH, Pisinger C, Jørgensen T, Fenger RV, Linneberg A. Vitamin D status and chronic obstructive pulmonary disease: a prospective general population study PLoS One. 2014;9(3):e90654.
12. Shaheen SO, Jameson KA, Robinson SM, Boucher BJ, Syddall HE, Sayer AA, Cooper C, Holloway JW, Dennison EM. Relationship of vitamin D status to adult lung function and COPD. Thorax. 2011;66:692-8
13. Wright RJ, Black PN, Scragg R. Relationship between serum 25-hydroxyvitamin D and pulmonary function in the third national health and nutrition examination survey. Chest 2005;128:3792-3798.
14. Choi CJ, Seo M, Choi WS, Kim KS, Youn SA, Lindsey T, Choi YJ, Kim CM. Relationship between serum 25-hydroxyvitamin D and lung function among Korean adults in Korea National Health and Nutrition Examination Survey (KNHANES), 2008-2010. J Clin Endocrinol Metab. 2013;98(4):1703-10.
15. Lange NE, Saprrow D, Vokonas P, Litonjua AA. Vitamin D deficiency, smoking and lung function in the normative aging study. Am J Respir Crit Care Med 2012;186:612-21.
16. Kunisaki KM, Niewoehner DE, Singh RJ, Connett JE. Vitamin D status and longitudinal lung function decline in the Lung Health Study. Eur Respir J. 2011;37(2):238-43.
17. Schöttker B, Haug U, Schomburg L, Köhrle J, Perna L, Müller H, Holleczek B, Brenner H. Strong associations of 25-hydroxyvitamin D concentrations with all-cause, cardiovascular, cancer, and respiratory disease mortality in a large cohort study. Am J Clin Nutr. 2013;97(4):782-93.
18. Khaw KT, Luben R, Wareham N. Serum 25-hydroxyvitamin D, mortality, and incident cardiovascular disease, respiratory disease, cancers, and fractures: a 13-y prospective population study. Am J Clin Nutr. 2014;100(5):1361-70.
19. Chowdhury R, Kunutsor S, Vitezova A, Oliver-Williams C, Chowdhury S, Kiefte-de-Jong JC, Khan H, Baena CP, Prabhakaran D, Hoshen MB, Feldman BS, Pan A, Johnson L, Crowe F, Hu FB, Franco OH. Vitamin D and risk of cause specific death: systematic review and meta-analysis of observational cohort and randomised intervention studies. BMJ. 2014;348:g1903.
20. Persson LJ, Aanerud M, Hiemstra PS, Michelsen AE, Ueland T, Hardie JA, Aukrust P, Bakke PS, Eagan TM. Vitamin D, vitamin D binding protein, and longitudinal outcomes in COPD. PLoS One. 2015;10(3):e0121622.

For peer review only

21. Ford ES. Lung function, 25-hydroxyvitamin D concentrations and **mortality** in US adults. Eur J Clin Nutr. 2015;69:572-8.

22. Lee HM, Liu M, Lee K, Luo Y, Wong ND. Does low vitamin D amplify the association of COPD with total and cardiovascular disease mortality? Clin Cardiol. 2014;37(8):473-8.

23. Holmgaard DB, Mygind LH, Titlestad IL, Madsen H, Fruekilde PB, Pedersen SS, Pedersen C Serum vitamin D in patients with chronic obstructive lung disease does not correlate with mortality--results from a 10-year prospective cohort study.PLoS One. 2013;8(1):e53670.

24. Puhan MA, Siebeling L, Frei A, Zoller M, Bischoff-Ferrari H, ter Riet G. No association of 25-Hydroxyvitamin D with exacerbations in primary care Patients with COPD. Chest 2014;145:37-43.

25. Mattila T, Vasankari T, Rissanen H, Knekt P, Sares-Jaske L,Jaaskelainen T, Heliovaara M. Airway obstruction , serum vitamin D and mortality in a 33 year follow-up study. Eur J Clin Nutr 2019;73:1024-1032.

26. Færk G, Çolak Y, Afzal S, Nordestgaard BG.Færk G, et al. Low concentrations of 25-hydroxyvitamin D and long-term prognosis of COPD: a prospective cohort study. Eur J Epidemiol. 2018 Jun;33(6):567-577

27. Lennon LT, Ramsay SE, Papacosta O, Shaper AG, Wannamethee SG, Whincup PH. Cohort Profile Update: The British Regional Heart Study 1978-2014: 35 years follow-up of cardiovascular disease and ageing. Int J Epidemiol. 2015;44:826-826g.

28. Wannamethee SG, Welsh P, Papacosta O, Lennon L, Whincup PH, Sattar N. Elevated parathyroid hormone, but not vitamin D deficiency, is associated with increased risk of heart failure in older men with and without cardiovascular disease. Circ Heart Fail. 2014;7:732-9.

29. Wannamethee SG, Shaper AG, Papacosta O, Lennon L, Welsh P, Whincup PH Lung function and airway obstruction: associations with circulating markers of cardiac function and incident heart failure in older men-the British Regional Heart Study. Thorax. 2016;71(6):526-34.

30. Knox, S, Harris, J, Calton, L, Wallace, AM. A simple automated solid-phase extraction procedure for measurement of 25-hydroxyvitamin D3 and D2 by liquid chromatography-tandem mass spectrometry. Ann Clin Biochem 2009;46(Pt 3):226–230

For peer review only

31. Bouillon RA, Auwerx JH, Lissens WD, Pelemans WK . Vitamin D status in the elderly: seasonal substrate deficiency causes 1,25-dihydroxycholecalciferol deficiency. Am J Clin Nutr 1987;45:755–763.
32. Ross AC, Manson JE, Abrams SA, Aloia JF, Brannon PM, Clinton SK, Durazo-Arvizu RA, Gallagher JC, Gallo RL, Jones G, Kovacs CS, Mayne ST, Rosen CJ, Shapses SA. The 2011 report on dietary reference intakes for calcium and vitamin D from the Institute of Medicine: what clinicians need to know. J Clin Endocrinol Metab. 2011;96:53-8
33. Falaschetti E, Laiho J, Primatesta P, Purdon S. Prediction equations for normal and low lung function from the health Survey for England. Eur Respir J 2004;456-463.
34. Rabe KF, Hurd S, Anzueto A, Barnes PJ, Buist SA, Calverley P, Fukuchi Y, Jenkins C, Rodriguez-Roisin R, van Weel C, Zielinski J; Global Initiative for Chronic Obstructive Lung Disease. Global strategy for the diagnosis, management, and prevention of chronic obstructive pulmonary disease: GOLD executive summary. Am J Respir Crit Care Med. 2007; 176:532-55. Review.
35. Herr C, Greulich T, Koczulla RA, Meyer S, Zakharkina T, Branscheidt M, Eschmann R, Bals R. The role of vitamin D in pulmonary disease: COPD, asthma, infection, and cancer. Respir Res. 2011 Mar 18;12:31.
36. National Institute for Health and Care Excellence Clinical Knowledge Summaries *Vitamin D Deficiency in Adults* https://cks.nice.org.uk/topics/vitamin-d-deficiency-in-adults/(last revised April 2021)
37. National Institutes of Health *Vitamin D: Fact Sheet for Health Professionals: US Department of Health and Human Services*. https://ods.od.nih.gov/factsheets/VitaminD-HealthProfessional/ (last revised March 26, 2021).
38. Rosen CJ, Abrams SA, Aloia JF, Brannon PM, Clinton SK, Durazo-Arvizu RA, et al. IOM committee members respond to Endocrine Society vitamin D guidelines. J Clin Endocrinol Metab 2012; 97:1146-52.
39. Durup D, Jørgensen HL, Christensen J, Tjønneland A, Olsen A, Halkjær J, Lind B, Heegaard AM, Schwarz P A Reverse J-Shaped Association Between Serum 25-Hydroxyvitamin D and Cardiovascular Disease Mortality: The CopD Study. J Clin Endocrinol Metab. 2015; 100:2339-46.
40. Hansen JG, Gao W, Dupuis J, O'Connor GT, Tang W, Kowgier M, Sood A, Gharib SA, Palmer LJ, Fornage M, Heckbert SR, Psaty BM, Booth SL; SUNLIGHT

For peer review only

Consortium., Cassano PA Association of 25-Hydroxyvitamin D status and genetic variation in the vitamin D metabolic pathway with FEV1 in the Framingham Heart Study. Respir Res. 2015;16:81.

41. Lehouck A, Mathieu C, Carremans C, Baeke F, Verhaegen J, Van Eldere J, Decallonne B, Bouillon R, Decramer M, Janssens W. High doses of vitamin D to reduce exacerbations in chronic obstructive pulmonary disease: a randomized trial. Ann Intern Med. 2012;156(2):105-14.
42. Martineau AR, Jolliffe DA, Hooper RL, Greenberg L, Aloia JF, Bergman P, Dubnov-Raz G, Esposito S, Ganmaa D, Ginde AA, Goodall EC, Grant CC, Griffiths CJ, Janssens W, Laaksi I, Manaseki-Holland S, Mauger D, Murdoch DR, Neale R, Rees JR, Simpson S Jr, Stelmach I, Kumar GT, Urashima M, Camargo CA Jr. Vitamin D supplementation to prevent acute respiratory tract infections: systematic review and meta-analysis of individual participant data. BMJ. 2017;356: i6583.
43. Sethi S. Infection as a comorbidity of COPD. Eur Respir J. 2010 ;35:1209-15. Review.
44. Bjelakovic G, Gluud LL, Nikolova D, Whitfield K, Wetterslev J, Simonetti RG, Vitamin D supplementation for prevention of mortality in adults. Cochrane Database Syst Rev. 2014;1:CD007470. Review.

For peer review only

Table 1

Table 1 Baseline characteristics according to **25(OH)D** groups in men without history of heart failure

	25(OH)D (ng/mL)			*p-trend*
	<10 (N=363)	10-19 (N=1499)	≥20 (N=1713)	
Age (yrs)	69.6 (5.8)	68.7 (5.5)	68.4 (5.4)	*0.0003*
%smokers	25.3	12.8	9.5	*<0.0001*
% manual	56.2	52.7	54.9	*0.77*
% inactive	56.4	34.9	27.9	*<0.0001*
% heavy drinkers	6.1	2.9	3.7	*0.01*
%MI/stroke	18.2	13.6	13.4	*0.07*
% diabetes	16.5	14.1	11.0	*<0.0001*
% antihypertensive	38.6	32.0	31.6	*0.04*
% renal dysfunction	15.4	15.6	14.9	*0.63*
BMI (kg/m^2)	26.8 (4.5)	27.1 (3.8)	26.6 (3.3)	*0.007*
% obese	19.6	18.5	13.3	*<0.0001*
SBP (mmHg)	152.4 (26.1)	148.6 (23.9)	149.1 (23.6)	*0.16*
CRP (mg/L)*	2.25	1.65	1.57	*<0.0001*
IL-6	3.0	2.50	2.27	*<0.0001*
**Lung function**				
FEV1 (L)	2.35 (0.71)	2.58 (0.65)	2.67 (0.64)	*<0.0001*
% FEV predicted	80 (23)	86 (21)	89 (21)	*<0.0001*
Abnormal FEV1	12.3	4.9	3.9	*<0.0001*
FVC (L)	3.17 (0.90)	3.36 (0.85)	3.47 (0.83)	*<0.0001*
% FVC predicted	81 (20)	85 (19)	88 (19)	*<0.0001*
Abnormal FVC	9.1	5.5	4.1	*<0.0001*
FEV_1/FVC	0.74 (0.13)	0.77 (0.12)	0.77 (0.11)	*<0.0001*
%COPD	32.5	22.0	21.0	*<0.0001*
mild	6.3	6.5	7.8	*0.16*
Moderate	15.4	11.5	9.6	*0.001*
Severe	10.7	3.9	3.6	<0.0001
% restrictive lung disorder	28.7	30.2	26.5	0.07

Mean and SD unless specified; *Geometric mean and interquartile range

% refers to the % of men with the characteristics within the 25(OH)D groups.

For peer review only

Table 2

Restrictive and obstructive lung function patterns and cross-sectional associations with circulating 25(OH)D and vitamin D deficiency.

	Normal N=1756	Restrictive N=1011	Obstructive Mild N=254	Obstructive Moderate N=394	Obstructive Severe N=160
**FEV1,% predicted**	101%	75%	95%	66%	39%
**FVC,% predicted**	96%	67%	101%	83%	61%
**25OHD (ng/ml)**					
Mean (SD)	20.71 (0.21)	19.62 (0.29)	20.95 (0.61)	18.86 (0.81)	17.60 (0.74)
Adjusted difference in mean 25(OH)D (95%CI) *					
Age and season adjusted	0	-1.05 (-1.76,-0.32)	0.38 (-0.82,1.58)	-1.74 (-2.74,-0.74)	-2.86 (-3.36,-1.36)
Model 1	0	-0.69 (-1.38,0.00)	1.15 (-0.03,2.33)	-0.98 (-1.96,-0.01)	-1.51(-2.96,-0.06)
Model 2	0	-0.52 (-1.21,0.20)	1.00 (-0.18,2.18)	-0.85 (-1.85,0.16)	-1.21(-2.66,,0.24)
**% with vitamin D deficiency**	8.0	10.3	9.1	14.2	24.4
Relative odds (95%CI) of having vitamin D deficiency					
Age and season adjusted	1.00	1.36 (1.04,1.78)	0.99 (0.62,1.59)	1.82 (1.30,2.55)	3.52 (2.33,5.30)
Model 1	1.00	1.09 (0.82,1.45)	0.85 (0.51,1.40)	1.49 (1.04,2.14)	2.13 (1.35,3.35)
Model 2	1.00	1.01 (0.75,1.35)	0.89(0.54,1.48)	1.47 (1.03,2.11)	1.98 (1.26,3.14)

* difference in mean 25(OH)D compared to those with normal lung function

Model 1: adjusted for age, season, smoking, physical activity, social class, BMI, diabetes and pre-existing CVD

Model 2=Model 1+IL-6.

For peer review only

Table 3 Circulating 25(OH)D and adjusted hazards ratio (95%CI) for total CVD, respiratory and COPD mortality.

	25OHD (ng/ml)				
	<10 (deficient) (N=363)	10-19 (N=1499)	20+ (N=1713)	HR per 1 ng/ml increase in 25OHD	p-linear trend
**Total**					
Rate/1000	67.8 (287)	46.4 (988)	40.9 (1052)		
Age+season	1.80 (1.57,2.05)	1.16 (1.06,1.27)	1.00	0.983 (0.978,0.988)	<0.0001
Model 1	1.52 (1.32,1.75)	1.14 (1.04,1.25)	1.00	0.989 (0.984,0.994)	<0.0001
Model 2	1.56 (1.32,1.75)	1.14 (1.04,1.25)	1.00	0.989 (0.984,0.994)	<0.0001
Model 3	1.47 (1.28,1.70)	1.12 (1.02,1.22)	1.00	0.990 (0.985,0.995)	<0.0001
**CVD**					
Rate/1000	20.6 (87)	15.6 (332)	14.0 (359)		
Model 1	1.27 (0.99,1.64)	1.11 (0.95,1.30)	1.00		0.06
Model 2	1.28 (0.99,1.65)	1.11 (0.95,1.30)	1.00	0.992 (0.983,1.000)	0.06
Model 3	1.23 (0.95,1.59)	1.07 (0.92,1.26)	1.00	0.993 (0.985,1.002)	0.15
**Respiratory**					
Rate/1000	11.4 (48)	4.8 (103)	4.4 (113)		
Model 1	2.10 (1.46,3.02)	1.08 (0.82,1.43)	1.00	0.978 (0.963,0.993)	0.004
Model 2	2.07 (1.44,2.99)	1.08 (0.82,1.42)	1.00	0.978 (0.964,0.993)	0.005
Model 3	2.01 (1.39,2.90)	1.06 (0.80,1.40)	1.00	0.979 (0.964,0.994)	0.007
COPD					
Rate/1000	5.7 (24)	2.3 (48)	1.6 (42)		
Model 1	2.28 (1.32,3.91)	1.42 (0.92,2.17)	1.00	0.972 (0.950,0.994)	0.01
Model 2	2.25 (1.31,3.88)	1.43 (0.93,2.19)	1.00	0.972 (0.950,0.994)	0.01
Model 3	2.06 (1.19,3.58)	1.37 (0.88,2.11)	1.00	0.975 (0.953,0.997)	0.02

Model 1: adjusted for age, season, smoking, physical activity, social class, BMI, diabetes, use of antihypertensive treatment, pre-existing CVD and impaired lung function.

Model 2=Model 1+BMI

Model 3=Model 2+IL-6.

For peer review only

Table 4 Circulating 25(OH)D and adjusted hazards ratio (95%CI) for total mortality by patterns of lung function.

	25(OH)D (ng/ml)			
	<10 (deficient)	10-19	20+	*p-linear trend*
**None (N=1756)**				
Rate/1000 (n/N)	52.9 (100/141)	35.7 (397/716)	34.4 (490/899)	
Model 1	1.46 (1.16,1.84)	1.07 (0.93,1.23)	1.00	*0.02*
Model 2	1.45 (1.15,1.82)	1.06 (0.92,1.22)	1.00	*0.02*
Model 3	1.39 (1.10,1.75)	1.05 (0.91,1.22)	1.00	*0.04*
**Restrictive (N=1011)**				
Rate/1000 (n/N)	74.4 (86/104)	51.8 (341/453)	41.6 (305/454)	
Model 1	1.54 (1.18,2.00)	1.24 (1.05,1.46)	1.00	*0.002*
Model 2	1.52 (1.19,2.00)	1.27 (1.08,1.50)	1.00	*0.002*
Model 3	1.52 (1.17,1.98)	1.26 (1.07,1.49)	1.00	*0.002*
**Mild/Moderate COPD (N=648)**				
Rate/1000 (n/N)	75.3 (65/79)	56.7 (199/271)	49.0 (207/298)	
Model 1	1.68 (1.25,2.26)	1.18 (0.96,1.45)	1.00	*0.02*
Model 2	1.68 (1.25,2.25)	1.18 (0.96,1.45)	1.00	*0.04*
Model 3	1.58 (1.17,2.14)	1.11 (0.90,1.36)	1.00	*0.04*
**Severe COPD (N=160)**				
Rate/1000 (n/N)	96.4 (36/39)	71.8 (51/59)	65.0 (50/62)	
Model 1	1.41 (0.85,2.35)	0.87 (0.57,1.33)	1.00	*0.56*
Model 2	1.43 (0.85,2.38)	0.87 (0.57,1.34)	1.00	*0.56*
**Model 3**	1.39 (0.83,2.33)	0.85 (0.55,1.30)	1.00	*0.64*

Model 1: adjusted for age, season, smoking, physical activity, social class, diabetes, use of antihypertensive treatment and pre-existing CVD

Model 2=model 1+BMI

Model 3=Model 2+IL-6.

For peer review only

Table 5 Circulating 25(OH)D and adjusted hazard ratios (95%CI) for respiratory and COPD mortality by patterns of lung function

	25(OH)D (ng/ml)		
	<10 (deficient)	10-19	20+
**RESPIRATORY deaths**			
**None**			
Rates/1000 p-yrs	4.2 (8/141)	2.6 (29/716)	2.8 (40/899)
Adjusted+ HR	1.63 (0.74,3.62)	0.99 (0.60,1.62	1.00
**Restrictive**			
Rates/1000 p-yrs	11.6 (13/104)	5.8 (35/453)	4.9 (32/454)
Adjusted HR	2.34 (1.16,4.71)	1.26 (0.75,2.10)	1.00
**Mild/Moderate COPD**			
Rates/1000 p-yrs	15.0 (13/79)	6.8 (24/271)	6.2 (26/298)
Adjusted HR	2.91 (1.42,5.97)	1.16 (0.65,2.08)	1.00
**Severe COPD**			
Rates/1000 p-yrs	23.3 (14/39)	20.5 (15/59)	20.5 (15/62)
Adjusted HR	1.27 (0.53,3.03)	0.65 0.28,1.50)	1.00
**COPD deaths**			
**None**			
Rates/1000 p-yrs	1.0 (2/141)	0.4 (4/716)	0.4 (6/899)
Adjusted HR	4.78 (0.86,26.70)	1.13 (0.30,4.29)	1.00
**Restrictive**			
Rates/1000 p-yrs	2.7 (3/104)	2.8 (17/453)	0.9 (6/454)
Adjusted HR	3.26 (0.72,14.80)	3.64 (1.36,9.71)	1.00
**Mild/Moderate COPD**			
Rates/1000 p-yrs	11.4 (9/79)	4.9 (17/271)	3.9 (16/298)
Adjusted HR	3.75 (1.55,9.12)	1.46 (0.71,3.00)	1.00
**Severe COPD**			
Rates/1000 p-yrs	28.2 (10/39)	15.5 (10/59)	19.1 (14/62)
Adjusted HR	1.04 (0.40,2.73)	0.47 (0.18,1.26)	1.00

+Adjusted for age, season, smoking, physical activity, social class, diabetes, use of antihypertensive treatment, pre-existing CVD and BMI.

HR=hazard ratio

For peer review only

Supplementary Table S1 Lung function and adjusted hazard ratios (95%CI) for total mortality according to 5 groups of 25(OH)D.

			**25(OH)D (ng/ml)**		
	<10 (N=363) (N=363)	10-19 (N=1499)	29-29 (N=1207)	30-39 (N=403)	≥40 (N=103)
**Lung function**					
**Mean (Std)**					
FEV1(L)	2.35 (0.71)	2.58 (0.65)	2.68 (0.65)	2.66 (0.62)	2.64 (0.63)
FVC (L)	3.17 (0.90)	3.36 (0.85)	3.47 (0.83)	3.48 (0.81)	3.43 (0.88)
FEV1/FVC	0.74 (0.13)	0.77 (0.12)	0.77 (0.11)	0.77 (0.12)	0.77 (0.11)
%COPD	32.5	22.0	20.8	20.8	24.3
% Severe COPD	10.7	3.9	3.6	3.2	5.8
% Restrictive	28.7	30.2	26.8	25.3	28.1
**Total mortality**					
Rate/1000 per-yrs (n)	67.9 (287)	46.4 (988)	41.0 (740)	41.4 (251)	38.4 (61)
Hazard ratio (95%CI)					
Age+season adjusted	1.91 (1.44,2.53)	1.00	1.23 (0.95,1.59)	1.04 (0.79,1.36)	1.00 (0.76,1.33)
+Adjusted	1.52 (1.14,2.02)	1.00	1.14 (0.88,1.48)	1.01 (0.78,1.33)	0.97 (0.72,1.26)

+Adjusted for age, season, smoking, physical activity, social class, diabetes, use of antihypertensive treatment, pre-existng CVD and BMI.

For peer review only

STROBE Statement—checklist of items that should be included in reports of observational studies

	Item No	Recommendation	Page No
**Title and abstract**	1	(*a*) Indicate the study's design with a commonly used term in the title or the abstract	2
		(*b*) Provide in the abstract an informative and balanced summary of what was done and what was found	2
**Introduction**			
Background/rationale	2	Explain the scientific background and rationale for the investigation being reported	5
Objectives	3	State specific objectives, including any prespecified hypotheses	6
**Methods**			
Study design	4	Present key elements of study design early in the paper	6
Setting	5	Describe the setting, locations, and relevant dates, including periods of recruitment, exposure, follow-up, and data collection	6-7
Participants	6	(*a*) *Cohort study*—Give the eligibility criteria, and the sources and methods of selection of participants. Describe methods of follow-up<br>*Case-control study*—Give the eligibility criteria, and the sources and methods of case ascertainment and control selection. Give the rationale for the choice of cases and controls<br>*Cross-sectional study*—Give the eligibility criteria, and the sources and methods of selection of participants	6-8
		(*b*) *Cohort study*—For matched studies, give matching criteria and number of exposed and unexposed<br>*Case-control study*—For matched studies, give matching criteria and the number of controls per case	
Variables	7	Clearly define all outcomes, exposures, predictors, potential confounders, and effect modifiers. Give diagnostic criteria, if applicable	7-8
Data sources/ measurement	8*	For each variable of interest, give sources of data and details of methods of assessment (measurement). Describe comparability of assessment methods if there is more than one group	*7-9*
Bias	9	Describe any efforts to address potential sources of bias	15
Study size	10	Explain how the study size was arrived at	
Quantitative variables	11	Explain how quantitative variables were handled in the analyses. If applicable, describe which groupings were chosen and why	9
Statistical methods	12	(*a*) Describe all statistical methods, including those used to control for confounding	9
		(*b*) Describe any methods used to examine subgroups and interactions	9
		(*c*) Explain how missing data were addressed	9
		(*d*) *Cohort study*—If applicable, explain how loss to follow-up was addressed<br>*Case-control study*—If applicable, explain how matching of cases and controls was addressed<br>*Cross-sectional study*—If applicable, describe analytical methods taking account of sampling strategy	
		(*e*) Describe any sensitivity analyses	

Continued on next page

For peer review only - http://bmjopen.bmj.com/site/about/guidelines.xhtml

**Results**			
Participants	13*	(a) Report numbers of individuals at each stage of study—eg numbers potentially eligible, examined for eligibility, confirmed eligible, included in the study, completing follow-up, and analysed	6
		(b) Give reasons for non-participation at each stage	6
		(c) Consider use of a flow diagram	
Descriptive data	14*	(a) Give characteristics of study participants (eg demographic, clinical, social) and information on exposures and potential confounders	6-8
		(b) Indicate number of participants with missing data for each variable of interest	6
		(c) *Cohort study*—Summarise follow-up time (eg, average and total amount)	8
Outcome data	15*	*Cohort study*—Report numbers of outcome events or summary measures over time	*9*
		Case-control study—Report numbers in each exposure category, or summary measures of exposure	
		Cross-sectional study—Report numbers of outcome events or summary measures	
Main results	16	(*a*) Give unadjusted estimates and, if applicable, confounder-adjusted estimates and their precision (eg, 95% confidence interval). Make clear which confounders were adjusted for and why they were included	10-12
		(*b*) Report category boundaries when continuous variables were categorized	9
		(*c*) If relevant, consider translating estimates of relative risk into absolute risk for a meaningful time period	
Other analyses	17	Report other analyses done—eg analyses of subgroups and interactions, and sensitivity analyses	11
**Discussion**			
Key results	18	Summarise key results with reference to study objectives	12
Limitations	19	Discuss limitations of the study, taking into account sources of potential bias or imprecision. Discuss both direction and magnitude of any potential bias	15
Interpretation	20	Give a cautious overall interpretation of results considering objectives, limitations, multiplicity of analyses, results from similar studies, and other relevant evidence	12-15
Generalisability	21	Discuss the generalisability (external validity) of the study results	15
**Other information**			
Funding	22	Give the source of funding and the role of the funders for the present study and, if applicable, for the original study on which the present article is based	16

*Give information separately for cases and controls in case-control studies and, if applicable, for exposed and unexposed groups in cohort and cross-sectional studies.

Note: An Explanation and Elaboration article discusses each checklist item and gives methodological background and published examples of transparent reporting. The STROBE checklist is best used in conjunction with this article (freely available on the Web sites of PLoS Medicine at http://www.plosmedicine.org/, Annals of Internal Medicine at http://www.annals.org/, and Epidemiology at http://www.epidem.com/). Information on the STROBE Initiative is available at www.strobe-statement.org.

For peer review only - http://bmjopen.bmj.com/site/about/guidelines.xhtml

BMJ Open

Vitamin D deficiency, impaired lung function and total and respiratory mortality in a cohort of older men: Cross sectional and prospective findings from The British Regional Heart Study

Journal:	*BMJ Open*
Manuscript ID	bmjopen-2021-051560.R2
Article Type:	Original research
Date Submitted by the Author:	03-Nov-2021
Complete List of Authors:	Wannamethee, Goya; UCL Medical School, UCL Department of Primary Care & Population Health<br>Welsh, Paul; University of Glasgow, Institute of Cardiovascular and Medical Sciences<br>Papacosta, Olia; UCL Medical School, Primary Care and Population Health<br>Lennon, Lucy; University College London Medical School, Primary Care and Population Health<br>Whincup, Peter; St George's University of London
**Primary Subject Heading**:	Epidemiology
Secondary Subject Heading:	Respiratory medicine, Nutrition and metabolism
Keywords:	EPIDEMIOLOGY, RESPIRATORY MEDICINE (see Thoracic Medicine), NUTRITION & DIETETICS

SCHOLARONE™
Manuscripts

I, the Submitting Author has the right to grant and does grant on behalf of all authors of the Work (as defined in the below author licence), an exclusive licence and/or a non-exclusive licence for contributions from authors who are: i) UK Crown employees; ii) where BMJ has agreed a CC-BY licence shall apply, and/or iii) in accordance with the terms applicable for US Federal Government officers or employees acting as part of their official duties; on a worldwide, perpetual, irrevocable, royalty-free basis to BMJ Publishing Group Ltd ("BMJ") its licensees and where the relevant Journal is co-owned by BMJ to the co-owners of the Journal, to publish the Work in this journal and any other BMJ products and to exploit all rights, as set out in our licence.

The Submitting Author accepts and understands that any supply made under these terms is made by BMJ to the Submitting Author unless you are acting as an employee on behalf of your employer or a postgraduate student of an affiliated institution which is paying any applicable article publishing charge ("APC") for Open Access articles. Where the Submitting Author wishes to make the Work available on an Open Access basis (and intends to pay the relevant APC), the terms of reuse of such Open Access shall be governed by a Creative Commons licence – details of these licences and which Creative Commons licence will apply to this Work are set out in our licence referred to above.

Other than as permitted in any relevant BMJ Author's Self Archiving Policies, I confirm this Work has not been accepted for publication elsewhere, is not being considered for publication elsewhere and does not duplicate material already published. I confirm all authors consent to publication of this Work and authorise the granting of this licence.

For peer review only

Vitamin D deficiency, impaired lung function and total and respiratory mortality in a cohort of older men: Cross sectional and prospective findings from The British Regional Heart Study

S Goya Wannamethee[1], Paul Welsh[2], Olia Papacosta[1], Lucy Lennon[1], Peter H Whincup[3]

1.UCL Department of Primary Care & Population Health, UCL Medical School, Rowland Hill Street, London. NW3 2PF UK

2. Institute of Cardiovascular & Medical Sciences, BHF Glasgow Cardiovascular Research Centre, University of Glasgow

3. Population Health Research Institute, St George's, University of London, Cranmer Terrace, London. SW17 0RE, UK

Address for Correspondence Prof S Goya Wannamethee, Department Primary Care and Population Health, UCL Medical School, Royal Free Campus, Rowland Hill St, London NW32PF, UK.

e-mail g.wannamethee@ucl.ac.uk

Word count text: 3904

Tables 5

Supplementary Table 1

CONFLICT OF INTEREST NONE

For peer review only

ABSTRACT

Objectives Vitamin D deficiency is associated with chronic obstructive pulmonary disease (COPD). We examined the cross-sectional association between 25-hydroxyvitamin D [25(OH)D] and lung function impairment and assessed whether vitamin D deficiency is related to long term mortality in those with impaired lung function.

Design Prospective study

Setting General practices in the UK.

Participants 3575 men aged 60-79 years with no prevalent heart failure.

Outcome measures Airway obstruction and mortality. The Global Initiative on Obstructive Lung diseases (GOLD) spirometry criteria was used to define airway obstruction.

Results During the follow-up period of 20 years, there were 2327 deaths (114 COPD deaths). Vitamin D deficiency was defined as serum 25(OH)D levels <10 ng/ml; insufficiency as 25(OH)D 10-19 ng/mL; sufficient as 25(OH)D ≥20 ng/mL In cross-sectional analysis vitamin D deficiency was more prevalent in those with moderate COPD (FEV/FVC <70% and FEV1 50 to <80%) and severe COPD (FEV/FVC <70% and FEV1 < 50%) but not in those with mild COPD (FEV/FVC <70% and FEV1 ≥ 80%) or restrictive lung disease (FEV1/FVC ≥70% and FVC <80%) compared to men with normal lung function . Vitamin D deficiency was associated with increased risk of total and respiratory mortality in both men with COPD and men with restrictive lung disease after adjustment for confounders and inflammation. The adjusted hazard ratios (HRs) (95% CI) for total mortality comparing levels of 25(OH)D <10ng/mL to 25(OH)D >20 ng/ml were 1.39 (1.10,1.75), 1.52 (1.17,1.98), 1.58 (1.17,2.14) and 1.39 (0.83,2.33) for those with no lung impairment, restrictive lung function, mild/moderate COPD, and severe COPD respectively.

For peer review only

Conclusion Men with COPD were more likely to be vitamin D deficient than those with normal lung function. Vitamin D deficiency is associated with increased all-cause mortality in older men with no lung impairment as well as in those with restrictive or obstructive lung impairment.

Key words: Vitamin D deficiency, 25(OH)D; chronic obstructive lung disease; lung function; mortality

For peer review only

Strengths and limitations of this study

- This study is carried out in a high-risk group for vitamin D deficiency and COPD and examined restrictive and obstructive lung disease separately.

- The study population is socially representative of the UK.

- The study took into account a wide range of confounders and inflammatory markers that could explain the associations between vitamin D deficiency and lung function and mortality.

-. The study is limited by the use of a single measurement of vitamin D [25(OH)D].

- The study is restricted to a predominantly white male population of European extraction,

For peer review only

INTRODUCTION

Chronic obstructive lung disease (COPD) is a common chronic inflammatory disease in the elderly and is a major cause of morbidity and mortality [1]. Much attention has focussed on identifying factors which may influence prognosis and mortality in these patients. Vitamin D deficiency (ascertained from measuring the circulating 25-hydroxyvitamin D [25(OH)D] metabolite) is recognised as an important health problem particularly in the elderly [2] and has been associated with various diseases including respiratory infections, autoimmune disease, CVD, diabetes and cancer [3-6]. Although vitamin D deficiency is traditionally known for its role in bone health, there is growing interest in the role of vitamin D in the pathogenesis and severity of COPD [7]. Observational studies have found Vitamin D deficiency to be highly prevalent in patients with COPD [8-12]. While many cross-sectional population studies have associated higher 25(OH)D with better lung function as measured by the FEV1 [10-16], prospective studies of the association of baseline 25(OH)D with lung function decline and incident COPD have produced conflicting results [9-11,15,16]. Whether the association between 25(OH)D and impaired lung function is specific to COPD is also unclear. Vitamin D deficiency has been associated with increased mortality in the general population [17-19] but the associations with pulmonary death and total mortality in COPD patients remains uncertain. Longitudinal studies that have examined the association between 25(OH)D and mortality in COPD patients have mostly reported no significant association with mortality [20-24]. However, most of these previous studies have targeted moderate and severe COPD patients only. Two recent prospective studies showed that vitamin D deficiency or low vitamin D was associated with all-cause mortality in those with COPD in the general population [25,26] . Few studies have examined the association between vitamin D deficiency and impaired lung function in the general older population specifically and whether vitamin D deficiency is associated with mortality in older adults with milder degrees

For peer review only

of impaired lung function (restrictive or obstructive) has not been well studied. The aim of the present study is to 1) investigate the cross-sectional association between vitamin D deficiency and both restrictive and obstructive lung function impairment and 2) whether vitamin D deficiency is related to long term mortality in those with impaired lung function.

SUBJECTS AND METHODS

The British Regional Heart Study is a prospective study involving 7735 men aged 40-59 years drawn from one general practice in each of 24 British towns, who were screened between 1978 and 1980 [27]. The population studied was socio-economically representative of British men and comprises predominantly white Europeans (>99%). In 1998-2000, all surviving men, then aged 60-79 years, were invited for a 20th year follow-up examination. All men provided informed written consent. Ethical approval was obtained from the National Research Ethics Service Committee London. All men completed a mailed questionnaire providing information on their lifestyle and medical history, had a physical examination and provided a fasting blood sample. The samples were frozen and stored at -20^{0}C on the day of collection and transferred in batches for storage at -70°C until analysis, carried out after no more than one freeze-thaw cycle. The men were asked whether a doctor had ever told them that they had angina or myocardial infarction (MI), heart failure (HF) or stroke; details of their medications were recorded at the examination including use of bronchodilators (BNF code 3.1). 4252 men (77% of available survivors) attended for examination.

Study subjects

Blood measurements of 25(OH)D, were available in 3754 men (88%) at the 20-year follow-up examination (1998-2000). Of these men, we excluded 88 men with a history of a diagnosis of

For peer review only

HF as these men have exceptional mortality rates and reduced lung function and a further 91 men with no lung function measures leaving 3575 men for analysis.

Cardiovascular risk factor measurements at 1998-2000

Anthropometric measurements including body weight, height, and waist circumference (WC) were carried out. Details of measurement and classification methods for smoking status, physical activity, social class, alcohol intake, blood pressure and blood lipids in this cohort have been described [28,29]. Heavy drinking was defined as drinking more than five units (1 UK unit = 10g) of alcohol daily on most days. Physical activity scores were assigned on the basis of frequency and type of activity and the men were divided into 6 groups: none, occasional, light, moderate, moderately vigorous and vigorous. Subjects who reported none or occasional activity were classified as 'inactive'. On the basis of their reported cigarette smoking status the men were classified as never smokers, long term ex-smokers ($\geq$ 15 years) , recent ex-smokers (<15 years) and current smokers. Social class was derived from the longest held occupation recorded at the time of baseline questionnaire (1978-80) using the Registrar General's classification of occupations, with categories grouped as non-manual (I, II and III non-manual) and manual (III manual, IV and V). Interleukin-6 was assayed using a high-sensitivity ELISA (R & D Systems, Oxford, UK). C-reactive protein (CRP) was assayed by ultra-sensitive nephelometry (Dade Behring, Milton Keynes, UK).

Vitamin D

Measurement of 25(OH)D was performed on EDTA-anticoagulated plasma via a high-throughput method for the measurement of 25(OH)D_3 and -D_2 using a gold-standard liquid chromatography-tandem mass spectrometry method following an automated solid-phase extraction procedure [30,31]. Our method is calibrated and controlled using reagents from

For peer review only

Chromsystems GmbH (Manchester, UK) and is currently in routine clinical use. Results are reported as total 25(OH)D [$25OHD_2$ + $25OHD_3$); virtually all participants had an undetectable $25OHD_2$, which is commensurate with results observed in routine NHS use. The lower limit of sensitivity was 4ng/ml for both $25(OH)D_3$ and total 25(OH)D. Vitamin D deficiency was defined as 25(OH)D <10 ng/ml] and vitamin D insufficiency as 25(OH)D 10-19 ng/ml [2,31,32].

Lung function

Forced expiratory volume in 1 second (FEV1) and forced vital capacity (FVC) were measured using a Vitalograph Compact spirometer with the subject seated [29]. We used predictive equations for FEV1 and FVC that were derived from the general population Health Survey for England for males aged >25 years [33]. The following equations were used:

$$\text{predicted FEV1}=-\exp[-9.37674+0.000183*\text{age}-0.00011*\text{age}^2+\log(\text{height})]$$

$$\text{predicted FVC}= \exp[-10.36706+0.00434*\text{age}-0.00011*\text{age}^2+\log(\text{height})]$$

Predicted FEV1%=raw FEV1/predicted FEV1 and predicted FVC%=raw FVC/predicted FVC. We divided the men into 5 lung pattern groups according to their FEV1/FVC ratio using a modification of the criteria developed by The Global Initiative for Chronic Obstructive Lung Disease (GOLD) for defining the severity stages of obstructive airways [34]: 1) severe airflow obstruction (FEV1/FVC <0.70 and FEV1 <50% predicted), moderate airflow obstruction (FEV1/FVC <0.70 and FEV1 ≥ 50 to <80%), mild airflow obstruction (FEV1/FVC <0.70 and FEV1≥ 80%), restricted (FEV1/FVC ≥0.70 and FVC <80% predicted) and normal (FEV1/FVC ≥0.70 and FVC ≥80% and not on bronchodilators).

Follow-up

For peer review only

All men have been followed up from initial examination (1978-1980) for cardiovascular morbidity [27] and follow-up has been achieved for 99% of the cohort. In the present analyses, all-cause mortality is based on follow-up from re-examination in 1998-2000 at mean age 60-79 years to September 2019. Details on causes of deaths were collected through the National Health Service Central Register. COPD deaths were defined as deaths due to chronic bronchitis (ICD-9 codes 490-491), emphysema (ICD-9 code 492), and chronic airway obstruction (ICD-9 code 496).

For peer review only

Statistical analysis

The men were classified into 3 vitamin D groups: 25(OH)D <10ng/ml (deficient), 10-19 ng/ml (insufficient) and ≥ 20 ng/ml (sufficient). Cox's proportional hazards model was used to assess the multivariate-adjusted hazards ratio (relative risk) for mortality in a comparison of three vitamin D groups: <10ng/ml, 10-19 ng/ml and ≥20ng/ml and as well as in a 1 unit increase in 25OHD.

All analyses were initially adjusted for age and season. In the multivariate analyses we adjusted further for potential confounders known to be associated with lung function and mortality which included smoking, social class, physical activity, heavy drinking, BMI use of antihypertensive treatment, diabetes and pre-existing CVD. We also carried out supplementary analysis without inclusion of BMI since BMI may be a confounder or a potential mediator. We further adjusted for IL-6 as a potential mediator. In multivariate analyses, smoking, social class, physical activity, heavy drinking, diabetes, use of antihypertensive treatment and pre-existing CVD were fitted as categorical variables; IL-6 and BMI were fitted as a continuous variable. Analysis of variance was used to obtain adjusted mean differences in 25(OH)D between the lung impairment groups. Logistic regression was used to assess the relative odds of having

vitamin D deficiency (yes/no) for the lung impairment groups compared to those with normal lung function.

Patients and public involvement

Patients and the public were not involved in the conduct, design, analysis and interpretation of this research.

For peer review only

RESULTS

During the mean follow-up period of 20 years there were 2327 deaths from all causes in the 3575 men with no diagnosed HF, including 114 deaths due to COPD. The mean 25(OH)D was lower in men examined in winter (Dec-February) than in those examined in summer (June- August) 17.1 (7.4) vs 22.2 (9.4) ng/ml. The prevalence of COPD was also much higher in winter than in summer (30.2% vs 17%).

Baseline characteristics

Table 1 summarises baseline characteristics by the three 25(OH)D groups. At baseline, men with vitamin D deficiency had a more adverse health profile including the highest prevalence of smoking, heavy drinking, physical inactivity, obesity, hypertension and the highest mean CRP and IL-6 (markers of inflammation) (Table 1). 25.3% of those with vitamin D deficiency were smokers compared with only 9.5% in those who had vitamin D sufficiency. Lung function (both FEV and FVC) was higher in those with higher 25(OH)D levels. Men with vitamin D deficiency had the highest prevalence of COPD and this was seen for moderate and severe COPD but not for mild COPD. There was no association between vitamin D deficiency and prevalence of restrictive lung impairment. We divided the vitamin

D sufficient group [25(OH)D ≥20 ng/ml] further into those with ranges 20-29, 30-39 and those with ≥40 ng/ml. There was no further increase in lung function with increasing levels of 25(OH)D among the sufficient group (Supplementary Table 1).

Cross sectional associations between restrictive and obstructive lung function patterns and 25(OH)D and vitamin D deficiency

Table 2 shows the absolute mean 25(OH)D for the 5 lung function groups and the adjusted difference in mean 25(OH)D for those with impaired lung function compared to those with normal lung function. Mean 25(OH)D was lower in men with moderate or severe airway obstruction but not in those with mild airway obstruction compared to those with normal function (Table 2). Men with moderate and severe COPD but not those with restrictive lung patterns were more likely to have vitamin D deficiency. Men with moderate and severe COPD showed increased odds of having vitamin D deficiency compared to those with normal lung function even after adjustment for potential confounders including age, season, smoking, physical activity, social class, heavy drinking, BMI, diabetes, and pre-existing CVD (Table 2). These associations persisted after further adjustment for IL-6.

Prospective associations between 25(OHD) and Total mortality

25(OH)D was inversely associated with total mortality even after adjustment for potential confounders, BMI and inflammation (IL-6) (Table 3). The inverse association persisted upon exclusion of current smokers. Compared to those with vitamin D sufficiency the adjusted HR (95%CI) were 1.41 (1.19,1.68) and 1.11 (1.00,1.24)] for those with vitamin D deficiency and insufficiency respectively (p=0.002 for trend). The inverse association was seen for mortality

For peer review only

from respiratory and COPD causes but not from CVD causes after adjustment for potential confounders, BMI and inflammation (Table 3).

When examined by lung function status, vitamin D deficiency was associated with increased mortality in those with normal lung function as well as in those with restrictive lung disorder or mild/moderate COPD even after adjustment for confounders and inflammation (Table 4). In men with severe COPD a statistically non- significant increase in mortality was seen possibly due to small numbers. Vitamin D deficiency was associated with increased risk of respiratory mortality even after adjustment although this was statistically significant only in the restrictive and mild and moderate COPD group possibly because of small numbers (Table 5). The number of COPD deaths was small, but vitamin D deficiency was associated with increased risk of COPD mortality in all groups with the exception of those with severe COPD.

Discussion

Our findings confirm previous observational population studies on the association between low 25(OH)D and COPD and total mortality [13-19] and extend these findings further. The association was not explained by smoking, physical inactivity, or inflammation (IL-6) which are known to relate to both COPD and vitamin D deficiency [35]. Restrictive lung function, by contrast, was not associated with vitamin D deficiency. The definition of vitamin D deficiency and insufficiency remains controversial; the United Kingdom's National Institute for Health and Care Excellence defines it as <25 nmol/l (<10 ng/ml) [36], while the United States' Institute of Medicine defines it as <30 nmol/l (<12.5 ng/ml) [37]. We have shown that 25(OH)D <10ng/ml which is regarded as deficient [32] to be associated with increased risk of

For peer review only

total mortality and COPD mortality in those with restrictive and mild/moderate COPD but not in those with severe COPD which was independent of known potential confounders.

25(OH)D levels of 50 nmol/L (20 ng/mL) or more are sufficient for most people [37]. In contrast, the Endocrine Society stated that, for clinical practice, a serum 25(OH)D concentration of more than 75 nmol/L (30 ng/mL) is necessary to maximize the effect of vitamin D on calcium, bone, and muscle metabolism [38]. When those with 25(OH)D concentration >20 ng/ml were divided further there was no evidence that those with levels 20-29 ng/ml had lower lung function or higher mortality than those with levels >30 ng/ml. There has been suggestion that mortality is also increased in those with higher extreme 25(OH)D levels (>100 nmol/l or 40ng/ml) [39]. The number of older men with levels $\geq$ 40 ng/ml in this study was small and we observed no excess mortality in this group in contrast to the Copenhagen Vitamin D Study where the average age (50 years) was younger [39].

Vitamin D deficiency and COPD

It is now established that vitamin D deficiency (variously defined) is highly prevalent in patients with COPD [6,7,8,9,12]. In line with these studies, we have shown in cross sectional analysis that those with COPD were more likely to have vitamin D deficiency (<10ng/ml) than those with normal lung function and the prevalence of vitamin D deficiency increased with increasing severity of COPD even after taking into account a wide range of possible confounders and mediators including smoking, physical inactivity, BMI, and inflammation (IL-6). Whether vitamin D deficiency is a consequence of COPD or whether it plays a role in the development of COPD is still unclear [7]. Despite the biological evidence that vitamin D may improve lung function through its action on regulating inflammation, inducing antimicrobial peptides and/ or

For peer review only

its action on muscle [6,35], the potential for reverse causality and confounding remains problematic.

Prospective population studies on vitamin D [25(OH)D)]and lung function decline or incident COPD have yielded inconsistent results, with some reporting vitamin D deficiency or low vitamin D [25(OH)D] to be associated with a greater decline in lung function or increased risk of COPD compared to those with sufficient levels [10,15,20] while others report null findings [11,16]. Small-scale genetic studies on polymorphisms in the vitamin D binding protein and rate of decline in lung function are also inconclusive [9,40]. Although we did not have incident COPD, we have shown that vitamin D deficiency (25OHD <10 ng/ml) was related to COPD mortality in all men except those with severe COPD; this was not explained by potential confounders and persisted after taking IL-6 into account. Our findings that COPD mortality in those with mild/moderate COPD is significantly increased only in those with vitamin D deficiency is consistent with findings from a randomised control trial (RCT) in subjects with COPD in which a post-hoc analysis was suggestive of reductions in exacerbations in the vitamin D supplementation group when subjects had what was regarded as severe vitamin D deficiency (25OHD <10ng/mL) only [41].

It is possible that vitamin D does not play a direct role in the progression of COPD but may prevent the development of COPD and COPD deaths through its prevention of respiratory tract infections. Evidence from both observational and RCTs have shown that vitamin D supplementation prevents respiratory tract infections [42] which are a frequent complication of COPD and have been implicated in the onset and progression and exacerbation of chronic lung disease [43]. The lack of association with COPD deaths in those with severe COPD may be due

For peer review only - http://bmjopen.bmj.com/site/about/guidelines.xhtml

to the advancement of the disease which may not be viable to detect any benefits of vitamin D. Alternately this may be due to lack of power as 25(OH)D levels in these patients are low and relatively few patients have sufficient 25(OH)D levels.

Vitamin D and total mortality in COPD subjects

Relatively few studies have examined 25(OH)D (vitamin D) and mortality in subjects with COPD and of those that have, many have reported null results and most have targeted moderate to very severe COPD patients [20,21,23,24]. Two of the population- based studies that have examined the association between 25(OH)D and mortality stratified by lung function status did not show low 25(OH)D (variously defined) to be independently associated with increased mortality [21,22]. However, in both these studies those with severe COPD were included and vitamin D deficiency [25(OH)D <10 ng/ml] specifically was not examined. Thus, the null findings with mortality in previous reports may be due to either the inclusion of those with severe COPD who are at an advanced stage of the disease, in whom vitamin D treatment will have little effect and /or the levels of 25(OH)D studied. However, two recent prospective studies have shown that 25(OH)D or low 25(OH)D (defined as <12.5 nmol/l) was associated with all- cause mortality in those with COPD in the general population [25,26]. A recent Cochrane review of vitamin D supplementation trials provided evidence that vitamin D_3 supplementation seem to decrease mortality in elderly adults [44].

Strengths and limitations

This study is based on a cohort of older (60–79-year-old) men who constitute a high-risk group for vitamin D deficiency and COPD. The study population is socially representative

For peer review only

of the UK, and follow-up rates in the British Regional Heart Study are exceptionally high. We were able to take into account a wide range of confounders and inflammatory markers. Our biochemical assays were based on routine clinical assays and are therefore robust. However, blood measurements were based on a single measurement, raising the possibility that the strengths of associations may have been underestimated. In addition, we cannot preclude the possibility of residual confounding. It was based on an older predominantly white male population of European extraction, so that the results cannot be generalized directly to women, younger populations, or other ethnic groups. Like most other epidemiological studies, we did not have lung function post bronchodilator. Although the present study was a prospective observational study and not a randomised trial, the association between vitamin D deficiency and mortality appeared independent of confounding factors and inflammation.

Conclusion

Men with COPD but not men with restrictive lung function were more likely to be vitamin D deficient than those with normal lung function. Vitamin D deficiency was associated with increased total mortality and mortality from respiratory and COPD causes in older men, and this was seen in those without lung impairment and in those with earlier stages of lung impairment. Intervention trials in older people with mild or moderate impaired lung function are needed to confirm whether increasing vitamin D levels through supplements in those with vitamin D deficiency will reduce both risk of COPD deaths and overall mortality.

DATA SHARING STATEMENT

For peer review only

The data that support the findings of this study are available from the corresponding author upon reasonable request (contact Lucy Lennon at l.lennon@ucl.ac.uk).

ETHICS APPROVAL STATEMENT

All men provided informed written consent. Ethical approval was obtained from the National Research Ethics Service Committee London (MREC 02/2/91 ID191747).

FUNDING SOURCES The British Regional Heart Study is a Research Group supported by the British Heart Foundation (BHF) Programme grant (RG/19/4/34452).

AUTHORS' CONTRIBUTORSHIP STATEMENT

SGW initiated the concept and design of the paper, analysed the data and drafted the manuscript. PW and PHW contributed to the interpretation of data. OP contributed to the analysis of the paper. PW, LL and PHW contributed to the acquisition of the data. All authors revised it critically for important intellectual content and approved the final version of the manuscript.

For peer review only

References

1. Rabe KF, Watz H. Chronic obstructive pulmonary disease. Lancet 2017;389:1931-1940.
2. Holick MF. Vitamin D deficiency . N Eng J Med 2007;357:266-281.
3. Brøndum-Jacobsen P, Benn M, Jensen GB, Nordestgaard BG. 25-hydroxyvitamin d levels and risk of ischemic heart disease, myocardial infarction, and early death: population-based study and meta-analyses of 18 and 17 studies. Arterioscler Thromb Vasc Biol. 2012 Nov;32(11):2794-802.
4. Forouhi NG, Ye Z, Rickard AP, Khaw KT, Luben R, Langenberg C, Wareham NJ. Circulating 25-hydroxyvitamin D concentration and the risk of type 2 diabetes: results from the European Prospective Investigation into Cancer (EPIC)-Norfolk cohort and updated meta-analysis of prospective studies. Diabetologia. 2012;55(8):2173-82.
5. Gandini S, Boniol M, Haukka J, Byrnes G, Cox B, Sneyd MJ, Mullie P, Autier P. Meta-analysis of observational studies of serum 25-hydroxyvitamin D levels and colorectal, breast and prostate cancer and colorectal adenoma. Int J Cancer. 2011;128:1414-24.
6. Ginde AA, Mansbach JM, Camargo CA Jr. Association between serum 25-hydroxyvitamin D level and upper respiratory tract infection in the Third National Health and Nutrition Examination Survey. Arch Intern Med. 2009;169:384-90.
7. Janssens W, Decramer M, Mathieu C, Korf H Vitamin D and chronic obstructive pulmonary disease: hype or reality? Lancet Respir Med. 2013;1(10):804-12.
8. Zhu M, Wang T, Wang C, Ji Y. The association between vitamin D and COPD risk, severity, and exacerbation: an updated systematic review and meta-analysis. Int J COPD 2016:11 2597-2607.
9. Persson LJ, Aanerud M, Hiemstra PS, Hardie JA, Bakke PS, Eagan TM. Chronic obstructive pulmonary disease is associated with low levels of vitamin D. PLoS One. 2012;7(6):e38934.
10. Afzal S, Lange P, Bojesen SE, Freiberg JJ, Nordestgaard BG. Plasma 25-hydroxyvitamin D, lung function and risk of chronic obstructive pulmonary disease. Thorax 2014;69:24-31.

For peer review only

11. Skaaby T, Husemoen LL, Thuesen BH, Pisinger C, Jørgensen T, Fenger RV, Linneberg A. Vitamin D status and chronic obstructive pulmonary disease: a prospective general population study PLoS One. 2014;9(3):e90654.
12. Shaheen SO, Jameson KA, Robinson SM, Boucher BJ, Syddall HE, Sayer AA, Cooper C, Holloway JW, Dennison EM. Relationship of vitamin D status to adult lung function and COPD. Thorax. 2011;66:692-8
13. Wright RJ, Black PN, Scragg R. Relationship between serum 25-hydroxyvitamin D and pulmonary function in the third national health and nutrition examination survey. Chest 2005;128:3792-3798.
14. Choi CJ, Seo M, Choi WS, Kim KS, Youn SA, Lindsey T, Choi YJ, Kim CM. Relationship between serum 25-hydroxyvitamin D and lung function among Korean adults in Korea National Health and Nutrition Examination Survey (KNHANES), 2008-2010. J Clin Endocrinol Metab. 2013;98(4):1703-10.
15. Lange NE, Saprrow D, Vokonas P, Litonjua AA. Vitamin D deficiency, smoking and lung function in the normative aging study. Am J Respir Crit Care Med 2012;186:612-21.
16. Kunisaki KM, Niewoehner DE, Singh RJ, Connett JE. Vitamin D status and longitudinal lung function decline in the Lung Health Study. Eur Respir J. 2011;37(2):238-43.
17. Schöttker B, Haug U, Schomburg L, Köhrle J, Perna L, Müller H, Holleczek B, Brenner H. Strong associations of 25-hydroxyvitamin D concentrations with all-cause, cardiovascular, cancer, and respiratory disease mortality in a large cohort study. Am J Clin Nutr. 2013;97(4):782-93.
18. Khaw KT, Luben R, Wareham N. Serum 25-hydroxyvitamin D, mortality, and incident cardiovascular disease, respiratory disease, cancers, and fractures: a 13-y prospective population study. Am J Clin Nutr. 2014;100(5):1361-70.
19. Chowdhury R, Kunutsor S, Vitezova A, Oliver-Williams C, Chowdhury S, Kiefte-de-Jong JC, Khan H, Baena CP, Prabhakaran D, Hoshen MB, Feldman BS, Pan A, Johnson L, Crowe F, Hu FB, Franco OH. Vitamin D and risk of cause specific death: systematic review and meta-analysis of observational cohort and randomised intervention studies. BMJ. 2014;348:g1903.
20. Persson LJ, Aanerud M, Hiemstra PS, Michelsen AE, Ueland T, Hardie JA, Aukrust P, Bakke PS, Eagan TM. Vitamin D, vitamin D binding protein, and longitudinal outcomes in COPD. PLoS One. 2015;10(3):e0121622.

For peer review only

21. Ford ES. Lung function, 25-hydroxyvitamin D concentrations and **mortality** in US adults. Eur J Clin Nutr. 2015;69:572-8.
22. Lee HM, Liu M, Lee K, Luo Y, Wong ND. Does low vitamin D amplify the association of COPD with total and cardiovascular disease mortality? Clin Cardiol. 2014;37(8):473-8.
23. Holmgaard DB, Mygind LH, Titlestad IL, Madsen H, Fruekilde PB, Pedersen SS, Pedersen C Serum vitamin D in patients with chronic obstructive lung disease does not correlate with mortality--results from a 10-year prospective cohort study.PLoS One. 2013;8(1):e53670.
24. Puhan MA, Siebeling L, Frei A, Zoller M, Bischoff-Ferrari H, ter Riet G. No association of 25-Hydroxyvitamin D with exacerbations in primary care Patients with COPD. Chest 2014;145:37-43.
25. Mattila T, Vasankari T, Rissanen H, Knekt P, Sares-Jaske L,Jaaskelainen T, Heliovaara M. Airway obstruction , serum vitamin D and mortality in a 33 year follow-up study. Eur J Clin Nutr 2019;73:1024-1032.
26. Færk G, Çolak Y, Afzal S, Nordestgaard BG.Færk G, et al. Low concentrations of 25-hydroxyvitamin D and long-term prognosis of COPD: a prospective cohort study. Eur J Epidemiol. 2018 Jun;33(6):567-577
27. Lennon LT, Ramsay SE, Papacosta O, Shaper AG, Wannamethee SG, Whincup PH. Cohort Profile Update: The British Regional Heart Study 1978-2014: 35 years follow-up of cardiovascular disease and ageing. Int J Epidemiol. 2015;44:826-826g.
28. Wannamethee SG, Welsh P, Papacosta O, Lennon L, Whincup PH, Sattar N. Elevated parathyroid hormone, but not vitamin D deficiency, is associated with increased risk of heart failure in older men with and without cardiovascular disease. Circ Heart Fail. 2014;7:732-9.
29. Wannamethee SG, Shaper AG, Papacosta O, Lennon L, Welsh P, Whincup PH Lung function and airway obstruction: associations with circulating markers of cardiac function and incident heart failure in older men-the British Regional Heart Study. Thorax. 2016;71(6):526-34.
30. Knox, S, Harris, J, Calton, L, Wallace, AM. A simple automated solid-phase extraction procedure for measurement of 25-hydroxyvitamin D3 and D2 by liquid chromatography-tandem mass spectrometry. Ann Clin Biochem 2009;46(Pt 3):226–230

For peer review only

31. Bouillon RA, Auwerx JH, Lissens WD, Pelemans WK . Vitamin D status in the elderly: seasonal substrate deficiency causes 1,25-dihydroxycholecalciferol deficiency. Am J Clin Nutr 1987;45:755–763.
32. Ross AC, Manson JE, Abrams SA, Aloia JF, Brannon PM, Clinton SK, Durazo-Arvizu RA, Gallagher JC, Gallo RL, Jones G, Kovacs CS, Mayne ST, Rosen CJ, Shapses SA. The 2011 report on dietary reference intakes for calcium and vitamin D from the Institute of Medicine: what clinicians need to know. J Clin Endocrinol Metab. 2011;96:53-8
33. Falaschetti E, Laiho J, Primatesta P, Purdon S. Prediction equations for normal and low lung function from the health Survey for England. Eur Respir J 2004;456-463.
34. Rabe KF, Hurd S, Anzueto A, Barnes PJ, Buist SA, Calverley P, Fukuchi Y, Jenkins C, Rodriguez-Roisin R, van Weel C, Zielinski J; Global Initiative for Chronic Obstructive Lung Disease. Global strategy for the diagnosis, management, and prevention of chronic obstructive pulmonary disease: GOLD executive summary. Am J Respir Crit Care Med. 2007; 176:532-55. Review.
35. Herr C, Greulich T, Koczulla RA, Meyer S, Zakharkina T, Branscheidt M, Eschmann R, Bals R. The role of vitamin D in pulmonary disease: COPD, asthma, infection, and cancer. Respir Res. 2011 Mar 18;12:31.
36. National Institute for Health and Care Excellence Clinical Knowledge Summaries *Vitamin D Deficiency in Adults* https://cks.nice.org.uk/topics/vitamin-d-deficiency-in-adults/(last revised April 2021)
37. National Institutes of Health *Vitamin D: Fact Sheet for Health Professionals: US Department of Health and Human Services*. https://ods.od.nih.gov/factsheets/VitaminD-HealthProfessional/ (last revised March 26, 2021).
38. Rosen CJ, Abrams SA, Aloia JF, Brannon PM, Clinton SK, Durazo-Arvizu RA, et al. IOM committee members respond to Endocrine Society vitamin D guidelines. J Clin Endocrinol Metab 2012; 97:1146-52.
39. Durup D, Jørgensen HL, Christensen J, Tjønneland A, Olsen A, Halkjær J, Lind B, Heegaard AM, Schwarz P A Reverse J-Shaped Association Between Serum 25-Hydroxyvitamin D and Cardiovascular Disease Mortality: The CopD Study. J Clin Endocrinol Metab. 2015; 100:2339-46.
40. Hansen JG, Gao W, Dupuis J, O'Connor GT, Tang W, Kowgier M, Sood A, Gharib SA, Palmer LJ, Fornage M, Heckbert SR, Psaty BM, Booth SL; SUNLIGHT

For peer review only

Consortium., Cassano PA Association of 25-Hydroxyvitamin D status and genetic variation in the vitamin D metabolic pathway with FEV1 in the Framingham Heart Study. Respir Res. 2015;16:81.

41. Lehouck A, Mathieu C, Carremans C, Baeke F, Verhaegen J, Van Eldere J, Decallonne B, Bouillon R, Decramer M, Janssens W. High doses of vitamin D to reduce exacerbations in chronic obstructive pulmonary disease: a randomized trial. Ann Intern Med. 2012;156(2):105-14.
42. Martineau AR, Jolliffe DA, Hooper RL, Greenberg L, Aloia JF, Bergman P, Dubnov-Raz G, Esposito S, Ganmaa D, Ginde AA, Goodall EC, Grant CC, Griffiths CJ, Janssens W, Laaksi I, Manaseki-Holland S, Mauger D, Murdoch DR, Neale R, Rees JR, Simpson S Jr, Stelmach I, Kumar GT, Urashima M, Camargo CA Jr. Vitamin D supplementation to prevent acute respiratory tract infections: systematic review and meta-analysis of individual participant data. BMJ. 2017;356: i6583.
43. Sethi S. Infection as a comorbidity of COPD. Eur Respir J. 2010 ;35:1209-15. Review.
44. Bjelakovic G, Gluud LL, Nikolova D, Whitfield K, Wetterslev J, Simonetti RG, Vitamin D supplementation for prevention of mortality in adults. Cochrane Database Syst Rev. 2014;1:CD007470. Review.

For peer review only

Table 1

Table 1 Baseline characteristics according to **25(OH)D** groups in men without history of heart failure

				p-trend
		25(OH)D (ng/mL)		
	<10 (N=363)	10-19 (N=1499)	≥20 (N=1713)	
Age (yrs)	69.6 (5.8)	68.7 (5.5)	68.4 (5.4)	*0.0003*
%smokers	25.3	12.8	9.5	*<0.0001*
% manual	56.2	52.7	54.9	*0.77*
% inactive	56.4	34.9	27.9	*<0.0001*
% heavy drinkers	6.1	2.9	3.7	*0.01*
%MI/stroke	18.2	13.6	13.4	*0.07*
% diabetes	16.5	14.1	11.0	*<0.0001*
% antihypertensive	38.6	32.0	31.6	*0.04*
% renal dysfunction	15.4	15.6	14.9	*0.63*
BMI (kg/m^2)	26.8 (4.5)	27.1 (3.8)	26.6 (3.3)	*0.007*
% obese	19.6	18.5	13.3	*<0.0001*
SBP (mmHg)	152.4 (26.1)	148.6 (23.9)	149.1 (23.6)	*0.16*
CRP (mg/L)*	2.25	1.65	1.57	*<0.0001*
IL-6	3.0	2.50	2.27	*<0.0001*
**Lung function**				
FEV1 (L)	2.35 (0.71)	2.58 (0.65)	2.67 (0.64)	*<0.0001*
% FEV predicted	80 (23)	86 (21)	89 (21)	*<0.0001*
Abnormal FEV1	12.3	4.9	3.9	*<0.0001*
FVC (L)	3.17 (0.90)	3.36 (0.85)	3.47 (0.83)	*<0.0001*
% FVC predicted	81 (20)	85 (19)	88 (19)	*<0.0001*
Abnormal FVC	9.1	5.5	4.1	*<0.0001*
FEV_1/FVC	0.74 (0.13)	0.77 (0.12)	0.77 (0.11)	*<0.0001*
%COPD	32.5	22.0	21.0	*<0.0001*
mild	6.3	6.5	7.8	*0.16*
Moderate	15.4	11.5	9.6	*0.001*
Severe	10.7	3.9	3.6	<0.0001
% restrictive lung disorder	28.7	30.2	26.5	0.07

Mean and SD unless specified; *Geometric mean and interquartile range

% refers to the % of men with the characteristics within the 25(OH)D groups.

For peer review only

Table 2

Restrictive and obstructive lung function patterns and cross-sectional associations with circulating 25(OH)D and vitamin D deficiency.

	Normal N=1756	Restrictive N=1011	Obstructive Mild N=254	Obstructive Moderate N=394	Obstructive Severe N=160
**FEV1,% predicted**	101%	75%	95%	66%	39%
**FVC,% predicted**	96%	67%	101%	83%	61%
**25OHD (ng/ml)**					
Mean (SD)	20.71 (0.21)	19.62 (0.29)	20.95 (0.61)	18.86 (0.81)	17.60 (0.74)
Adjusted difference in mean 25(OH)D (95%CI) *					
Age and season adjusted	0	-1.05 (-1.76,-0.32)	0.38 (-0.82,1.58)	-1.74 (-2.74,-0.74)	-2.86 (-3.36,-1.36)
Model 1	0	-0.69 (-1.38,0.00)	1.15 (-0.03,2.33)	-0.98 (-1.96,-0.01)	-1.51(-2.96,-0.06)
Model 2	0	-0.52 (-1.21,0.20)	1.00 (-0.18,2.18)	-0.85 (-1.85,0.16)	-1.21(-2.66,,0.24)
**% with vitamin D deficiency**	8.0	10.3	9.1	14.2	24.4
Relative odds (95%CI) of having vitamin D deficiency					
Age and season adjusted	1.00	1.36 (1.04,1.78)	0.99 (0.62,1.59)	1.82 (1.30,2.55)	3.52 (2.33,5.30)
Model 1	1.00	1.09 (0.82,1.45)	0.85 (0.51,1.40)	1.49 (1.04,2.14)	2.13 (1.35,3.35)
Model 2	1.00	1.01 (0.75,1.35)	0.89(0.54,1.48)	1.47 (1.03,2.11)	1.98 (1.26,3.14)

* difference in mean 25(OH)D compared to those with normal lung function

Model 1: adjusted for age, season, smoking, physical activity, social class, BMI, diabetes and pre-existing CVD

Model 2=Model 1+IL-6.

For peer review only

Table 3 Circulating 25(OH)D and adjusted hazards ratio (95%CI) for total CVD, respiratory and COPD mortality.

	25(OH)D (ng/ml)				
	<10 (deficient) (N=363)	10-19 (N=1499)	20+ (N=1713)	HR per 1 ng/ml increase in 25(OH)D	p-linear trend
**Total**					
Rate/1000	67.8 (287)	46.4 (988)	40.9 (1052)		
Age+season	1.80 (1.57,2.05)	1.16 (1.06,1.27)	1.00	0.983 (0.978,0.988)	<0.0001
Model 1	1.52 (1.32,1.75)	1.14 (1.04,1.25)	1.00	0.989 (0.984,0.994)	<0.0001
Model 2	1.56 (1.32,1.75)	1.14 (1.04,1.25)	1.00	0.989 (0.984,0.994)	<0.0001
Model 3	1.47 (1.28,1.70)	1.12 (1.02,1.22)	1.00	0.990 (0.985,0.995)	<0.0001
**CVD**					
Rate/1000	20.6 (87)	15.6 (332)	14.0 (359)		
Model 1	1.27 (0.99,1.64)	1.11 (0.95,1.30)	1.00	0.992(0.983,1.000)	0.06
Model 2	1.28 (0.99,1.65)	1.11 (0.95,1.30)	1.00	0.992 (0.983,1.000)	0.06
Model 3	1.23 (0.95,1.59)	1.07 (0.92,1.26)	1.00	0.993 (0.985,1.002)	0.15
**Respiratory**					
Rate/1000	11.4 (48)	4.8 (103)	4.4 (113)		
Model 1	2.10 (1.46,3.02)	1.08 (0.82,1.43)	1.00	0.978 (0.963,0.993)	0.004
Model 2	2.07 (1.44,2.99)	1.08 (0.82,1.42)	1.00	0.978 (0.964,0.993)	0.005
Model 3	2.01 (1.39,2.90)	1.06 (0.80,1.40)	1.00	0.979 (0.964,0.994)	0.007
COPD					
Rate/1000	5.7 (24)	2.3 (48)	1.6 (42)		
Model 1	2.28 (1.32,3.91)	1.42 (0.92,2.17)	1.00	0.972 (0.950,0.994)	0.01
Model 2	2.25 (1.31,3.88)	1.43 (0.93,2.19)	1.00	0.972 (0.950,0.994)	0.01
Model 3	2.06 (1.19,3.58)	1.37 (0.88,2.11)	1.00	0.975 (0.953,0.997)	0.02

Model 1: adjusted for age, season, smoking, physical activity, social class, diabetes, use of antihypertensive treatment, pre-existing CVD and impaired lung function.

Model 2=Model 1+BMI

Model 3=Model 2+IL-6.

For peer review only

Table 4 Circulating 25(OH)D and adjusted hazards ratio (95%CI) for total mortality by patterns of lung function.

	25(OH)D (ng/ml)			
	<10 (deficient)	10-19	20+	*p-linear trend*
**None (N=1756)**				
Rate/1000 (n/N)	52.9 (100/141)	35.7 (397/716)	34.4 (490/899)	
Model 1	1.46 (1.16,1.84)	1.07 (0.93,1.23)	1.00	*0.02*
Model 2	1.45 (1.15,1.82)	1.06 (0.92,1.22)	1.00	*0.02*
Model 3	1.39 (1.10,1.75)	1.05 (0.91,1.22)	1.00	*0.04*
**Restrictive (N=1011)**				
Rate/1000 (n/N)	74.4 (86/104)	51.8 (341/453)	41.6 (305/454)	
Model 1	1.54 (1.18,2.00)	1.24 (1.05,1.46)	1.00	*0.002*
Model 2	1.52 (1.19,2.00)	1.27 (1.08,1.50)	1.00	*0.002*
Model 3	1.52 (1.17,1.98)	1.26 (1.07,1.49)	1.00	*0.002*
**Mild/Moderate COPD (N=648)**				
Rate/1000 (n/N)	75.3 (65/79)	56.7 (199/271)	49.0 (207/298)	
Model 1	1.68 (1.25,2.26)	1.18 (0.96,1.45)	1.00	*0.02*
Model 2	1.68 (1.25,2.25)	1.18 (0.96,1.45)	1.00	*0.04*
Model 3	1.58 (1.17,2.14)	1.11 (0.90,1.36)	1.00	*0.04*
**Severe COPD (N=160)**				
Rate/1000 (n/N)	96.4 (36/39)	71.8 (51/59)	65.0 (50/62)	
Model 1	1.41 (0.85,2.35)	0.87 (0.57,1.33)	1.00	*0.56*
Model 2	1.43 (0.85,2.38)	0.87 (0.57,1.34)	1.00	*0.56*
**Model 3**	1.39 (0.83,2.33)	0.85 (0.55,1.30)	1.00	*0.64*

Model 1: adjusted for age, season, smoking, physical activity, social class, diabetes, use of antihypertensive treatment and pre-existing CVD

Model 2=model 1+BMI

Model 3=Model 2+IL-6.

For peer review only

Table 5 Circulating 25(OH)D and adjusted hazard ratios (95%CI) for respiratory and COPD mortality by patterns of lung function

	25(OH)D (ng/ml)		
	<10 (deficient)	10-19	20+
**RESPIRATORY deaths**			
**None**			
Rates/1000 p-yrs	4.2 (8/141)	2.6 (29/716)	2.8 (40/899)
Adjusted+ HR	1.63 (0.74,3.62)	0.99 (0.60,1.62	1.00
**Restrictive**			
Rates/1000 p-yrs	11.6 (13/104)	5.8 (35/453)	4.9 (32/454)
Adjusted HR	2.34 (1.16,4.71)	1.26 (0.75,2.10)	1.00
**Mild/Moderate COPD**			
Rates/1000 p-yrs	15.0 (13/79)	6.8 (24/271)	6.2 (26/298)
Adjusted HR	2.91 (1.42,5.97)	1.16 (0.65,2.08)	1.00
**Severe COPD**			
Rates/1000 p-yrs	23.3 (14/39)	20.5 (15/59)	20.5 (15/62)
Adjusted HR	1.27 (0.53,3.03)	0.65 0.28,1.50)	1.00
**COPD deaths**			
**None**			
Rates/1000 p-yrs	1.0 (2/141)	0.4 (4/716)	0.4 (6/899)
Adjusted HR	4.78 (0.86,26.70)	1.13 (0.30,4.29)	1.00
**Restrictive**			
Rates/1000 p-yrs	2.7 (3/104)	2.8 (17/453)	0.9 (6/454)
Adjusted HR	3.26 (0.72,14.80)	3.64 (1.36,9.71)	1.00
**Mild/Moderate COPD**			
Rates/1000 p-yrs	11.4 (9/79)	4.9 (17/271)	3.9 (16/298)
Adjusted HR	3.75 (1.55,9.12)	1.46 (0.71,3.00)	1.00
**Severe COPD**			
Rates/1000 p-yrs	28.2 (10/39)	15.5 (10/59)	19.1 (14/62)
Adjusted HR	1.04 (0.40,2.73)	0.47 (0.18,1.26)	1.00

+Adjusted for age, season, smoking, physical activity, social class, diabetes, use of antihypertensive treatment, pre-existing CVD and BMI.

HR=hazard ratio

For peer review only

Supplementary Table S1 Lung function and adjusted hazard ratios (95%CI) for total mortality according to 5 groups of 25(OH)D.

	**25(OH)D (ng/ml)**				
	<10 (N=363) (N=363)	10-19 (N=1499)	29-29 (N=1207)	30-39 (N=403)	≥40 (N=103)
**Lung function**					
**Mean (Std)**					
FEV1(L)	2.35 (0.71)	2.58 (0.65)	2.68 (0.65)	2.66 (0.62)	2.64 (0.63)
FVC (L)	3.17 (0.90)	3.36 (0.85)	3.47 (0.83)	3.48 (0.81)	3.43 (0.88)
FEV1/FVC	0.74 (0.13)	0.77 (0.12)	0.77 (0.11)	0.77 (0.12)	0.77 (0.11)
%COPD	32.5	22.0	20.8	20.8	24.3
% Severe COPD	10.7	3.9	3.6	3.2	5.8
% Restrictive	28.7	30.2	26.8	25.3	28.1
**Total mortality**					
Rate/1000 per-yrs (n)	67.9 (287)	46.4 (988)	41.0 (740)	41.4 (251)	38.4 (61)
Hazard ratio (95%CI)					
Age+season adjusted	1.91 (1.44,2.53)	1.00	1.23 (0.95,1.59)	1.04 (0.79,1.36)	1.00 (0.76,1.33)
+Adjusted	1.52 (1.14,2.02)	1.00	1.14 (0.88,1.48)	1.01 (0.78,1.33)	0.97 (0.72,1.26)

+Adjusted for age, season, smoking, physical activity, social class, diabetes, use of antihypertensive treatment, pre-existng CVD and BMI.

For peer review only

STROBE Statement—checklist of items that should be included in reports of observational studies

	Item No	Recommendation	Page No
**Title and abstract**	1	(*a*) Indicate the study's design with a commonly used term in the title or the abstract	2
		(*b*) Provide in the abstract an informative and balanced summary of what was done and what was found	2
**Introduction**			
Background/rationale	2	Explain the scientific background and rationale for the investigation being reported	5
Objectives	3	State specific objectives, including any prespecified hypotheses	6
**Methods**			
Study design	4	Present key elements of study design early in the paper	6
Setting	5	Describe the setting, locations, and relevant dates, including periods of recruitment, exposure, follow-up, and data collection	6-7
Participants	6	(*a*) *Cohort study*—Give the eligibility criteria, and the sources and methods of selection of participants. Describe methods of follow-up *Case-control study*—Give the eligibility criteria, and the sources and methods of case ascertainment and control selection. Give the rationale for the choice of cases and controls *Cross-sectional study*—Give the eligibility criteria, and the sources and methods of selection of participants	6-8
		(*b*) *Cohort study*—For matched studies, give matching criteria and number of exposed and unexposed *Case-control study*—For matched studies, give matching criteria and the number of controls per case	
Variables	7	Clearly define all outcomes, exposures, predictors, potential confounders, and effect modifiers. Give diagnostic criteria, if applicable	7-8
Data sources/ measurement	8*	For each variable of interest, give sources of data and details of methods of assessment (measurement). Describe comparability of assessment methods if there is more than one group	*7-9*
Bias	9	Describe any efforts to address potential sources of bias	15
Study size	10	Explain how the study size was arrived at	
Quantitative variables	11	Explain how quantitative variables were handled in the analyses. If applicable, describe which groupings were chosen and why	9
Statistical methods	12	(*a*) Describe all statistical methods, including those used to control for confounding	9
		(*b*) Describe any methods used to examine subgroups and interactions	9
		(*c*) Explain how missing data were addressed	9
		(*d*) *Cohort study*—If applicable, explain how loss to follow-up was addressed *Case-control study*—If applicable, explain how matching of cases and controls was addressed *Cross-sectional study*—If applicable, describe analytical methods taking account of sampling strategy	
		(*e*) Describe any sensitivity analyses	

Continued on next page

For peer review only - http://bmjopen.bmj.com/site/about/guidelines.xhtml

**Results**			
Participants	13*	(a) Report numbers of individuals at each stage of study—eg numbers potentially eligible, examined for eligibility, confirmed eligible, included in the study, completing follow-up, and analysed	6
		(b) Give reasons for non-participation at each stage	6
		(c) Consider use of a flow diagram	
Descriptive data	14*	(a) Give characteristics of study participants (eg demographic, clinical, social) and information on exposures and potential confounders	6-8
		(b) Indicate number of participants with missing data for each variable of interest	6
		(c) *Cohort study*—Summarise follow-up time (eg, average and total amount)	8
Outcome data	15*	*Cohort study*—Report numbers of outcome events or summary measures over time	*9*
		Case-control study—Report numbers in each exposure category, or summary measures of exposure	
		Cross-sectional study—Report numbers of outcome events or summary measures	
Main results	16	(*a*) Give unadjusted estimates and, if applicable, confounder-adjusted estimates and their precision (eg, 95% confidence interval). Make clear which confounders were adjusted for and why they were included	10-12
		(*b*) Report category boundaries when continuous variables were categorized	9
		(*c*) If relevant, consider translating estimates of relative risk into absolute risk for a meaningful time period	
Other analyses	17	Report other analyses done—eg analyses of subgroups and interactions, and sensitivity analyses	11
**Discussion**			
Key results	18	Summarise key results with reference to study objectives	12
Limitations	19	Discuss limitations of the study, taking into account sources of potential bias or imprecision. Discuss both direction and magnitude of any potential bias	15
Interpretation	20	Give a cautious overall interpretation of results considering objectives, limitations, multiplicity of analyses, results from similar studies, and other relevant evidence	12-15
Generalisability	21	Discuss the generalisability (external validity) of the study results	15
**Other information**			
Funding	22	Give the source of funding and the role of the funders for the present study and, if applicable, for the original study on which the present article is based	16

*Give information separately for cases and controls in case-control studies and, if applicable, for exposed and unexposed groups in cohort and cross-sectional studies.

Note: An Explanation and Elaboration article discusses each checklist item and gives methodological background and published examples of transparent reporting. The STROBE checklist is best used in conjunction with this article (freely available on the Web sites of PLoS Medicine at http://www.plosmedicine.org/, Annals of Internal Medicine at http://www.annals.org/, and Epidemiology at http://www.epidem.com/). Information on the STROBE Initiative is available at www.strobe-statement.org.

For peer review only
